# Human post-implantation blastocyst-like characteristics of Muse cells isolated from human umbilical cord

**DOI:** 10.1007/s00018-024-05339-4

**Published:** 2024-07-11

**Authors:** Yoshihiro Kushida, Yo Oguma, Kana Abe, Taichi Deguchi, Federico Girolamo Barbera, Noriyuki Nishimura, Kazumichi Fujioka, Sota Iwatani, Mari Dezawa

**Affiliations:** 1 Department of Stem Cell Biology and Histology, Tohoku University Graduate School of Medicine, 2-1 Seiryo-Machi, Aoba-Ku, Sendai, Miyagi 980-8575 Japan; 2 Department of Public Health, Kobe University Graduate School of Health Science, Kobe, Japan; 3 Department of Pediatrics, Kobe University Graduate School of Medicine, Kobe, Japan; 4 Department of Neonatology, Hyogo Prefectural Kobe Children’s Hospital, Kobe, Hyogo Japan

**Keywords:** Umbilical cord, Muse cells, XIST, Human post-implantation blastocyst, Single-cell RNA sequencing, DNA methylation

## Abstract

Muse cells, identified as cells positive for the pluripotent surface marker SSEA-3, are pluripotent-like endogenous stem cells located in the bone marrow (BM), peripheral blood, and organ connective tissues. The detailed characteristics of SSEA-3(+) cells in extraembryonic tissue, however, are unknown. Here, we demonstrated that similar to human-adult tissue-Muse cells collected from the BM, adipose tissue, and dermis as SSEA-3(+), human-umbilical cord (UC)-SSEA-3(+) cells express pluripotency markers, differentiate into triploblastic-lineage cells at a single cell level, migrate to damaged tissue, and exhibit low telomerase activity and non-tumorigenicity. Notably, ~ 20% of human-UC-SSEA-3(+) cells were negative for X-inactive specific transcript (XIST), a naïve pluripotent stem cell characteristic, whereas all human adult tissue-Muse cells are XIST-positive. Single-cell RNA sequencing revealed that the gene expression profile of human-UC-SSEA-3(+) cells was more similar to that of human post-implantation blastocysts than human-adult tissue-Muse cells. The DNA methylation level showed the same trend, and notably, the methylation levels in genes particularly related to differentiation were lower in human-UC-SSEA-3(+) cells than in human-adult tissue-Muse cells. Furthermore, human-UC-SSEA-3(+) cells newly express markers specific to extraembryonic-, germline-, and hematopoietic-lineages after differentiation induction in vitro whereas human-adult tissue-Muse cells respond only partially to the induction. Among various stem/progenitor cells in living bodies, those that exhibit properties similar to post-implantation blastocysts in a naïve state have not yet been found in humans. Easily accessible human-UC-SSEA-3(+) cells may be a valuable tool for studying early-stage human development and human reproductive medicine.

**Supplementary Information:**

The online version contains supplementary material available at 10.1007/s00018-024-05339-4.

## Introduction

Multilineage-differentiating stress enduring (Muse) cells, identified as cells positive for the pluripotent surface marker stage-specific embryonic antigen (SSEA)-3, are stress-tolerant pluripotent-like endogenous stem cells located in the bone marrow (BM), peripheral blood, and organ connective tissues [1–4]. Muse cells express pluripotency genes, including Nanog homeobox (NANOG), OCT3/4 (also known as POU class 5 homeobox 1 [POU5F1]), and SRY-box transcription factor 2 (SOX2) at moderate levels compared with embryonic stem cells (ESCs) and induced pluripotent stem (iPS) cells, and have the ability to self-renew and differentiate into triploblastic-lineages at a single-cell level [5]. Consistent with being endogenous to the body, they are non-tumorigenic, as supported by their low telomerase activity and the fact that they do not form teratomas when injected into the testes of SCID mice [1, 3, 6]. Maintenance of the pluripotency gene expression in Muse cells was recently shown to depend on the tumor suppressor microRNA let-7, rather than oncogenic Lin28, in contrast to ES and iPS cells [7].

Sphingosine-1-phosphate (S1P) is a general signal produced by damaged cells and is recognized by its receptor S1PR2 [8]. Endogenous or exogenously administered Muse cells, which express S1PR2, sense S1P and selectively migrate and home to the damaged tissue. Once there, they repair the tissue by engulfing damaged/apoptotic differentiated cells, recycling factors such as transcription factors that originally functioned in the differentiated cells, and rapidly differentiating into the same cell type as the engulfed damaged/apoptotic cells, thereby replacing the damaged tissue with healthy functioning cells [9, 10]. In addition, human leukocyte antigen (HLA)-mismatch allogenic Muse cells escape immune rejection and survive in the host tissue over the long term without immunosuppressant treatment; this characteristic may be partly explained by Muse cell expression of HLA-G, which contributes to the immune privilege of the placenta [10, 11]. Because of these characteristics, HLA-mismatch allogenic-Muse cell treatment does not require the introduction of genes to induce pluripotency or the induction of differentiation into target cell types before treatment and can be directly administered by intravenous injection without immunosuppressant treatment. Clinical trials evaluating the effects of Muse cell treatment by intravenous administration of donor-Muse cells without HLA-matching or long-term immunosuppressant treatment for several diseases have revealed the safety and therapeutic efficacy of this treatment in myocardial infarction [12], epidermolysis bullosa [13], subacute stroke [14], and amyotrophic lateral sclerosis [15].

Characterization of SSEA-3(+) cells from extraembryonic tissues such as the human umbilical cord (h-UC) [16–18], placenta [19], and amniotic membrane suggests the presence of cells equivalent to Muse cells in these tissues [6]. These cells express pluripotency genes and differentiate into triploblastic lineages, similar to Muse cells from adult tissues such as the BM, adipose tissue (AT), and skin [1–3]. In addition, human amniotic membrane-SSEA-3(+) cells are suggested to have the potential to differentiate into extraembryonic- and germline lineages [6]. Detailed analyses of the differentiation potential, gene expression, and DNA methylation status in extraembryonic tissue-SSEA-3(+) cells, however, have not yet been conducted.

In the present study, we examined the unique characteristics of h-UC-SSEA-3(+) cells that distinguish them from human adult tissue-Muse cells; h-UC-SSEA-3(+) cells exhibit post-implantation blastocyst-like properties as shown by their gene expression profile, DNA methylation status, and differentiation potential. A wide variety of somatic stem/progenitor cells, such as neural stem cells (NSCs), hematopoietic stem cells (HSCs), mesenchymal stem cells (MSCs), and adult tissue-Muse cells have been characterized [20, 21]. Among those, cells that exhibit characteristics similar to post-implantation blastocysts in naïve state, however, have not been reported in humans. The h-UC can be easily obtained as medical waste after birth without ethical problems or donor burden [22–25]. Easily accessible h-UC-SSEA-3(+) cells are a potentially valuable tool for studying early-stage human development and human reproductive medicine.

## Materials and methods

### Animals

C57BL/6 mice and CB17/Icr-Prkdc scid/CrlCrlj (SCID) mice were used in this study. All animals were treated according to the regulations of the Standards for Human Care and Use of Laboratory Animals of Tohoku University. The animal experiments were approved by the Animal Care and Experimentation Committee of Tohoku University Graduate School of Medicine (permission No. 2019MdA-265-03).

### Preparation of human Muse cells

The Ethics Review Board approved the use of h-UC according to the guidelines of the Ethics Committee of the Graduate School of Medicine, Tohoku University (approval number: 2020-1-293). The h-UCs were obtained from 5 infants delivered at 35 to 38 weeks of gestation with parental written consent at the Department of Pediatrics, Kobe University Graduate School of Medicine (approval number: 1370).

Human-UC-MSCs were first isolated from the entire h-UCs using the explant culture method [26]. Cells were maintained in culture medium comprising α-minimum essential medium (α-MEM, MilliporeSigma, St Louis, Mo, USA) with 10% (vol/vol) fetal bovine serum (FBS, HyClone, Logan, UT, USA), 1 ng/mL basic fibroblast growth factor (Miltenyi Biotec, Bergisch Gladbach, Germany), 2 mM GlutaMAX I (ThermoFisher Scientific, Waltham, MA, USA), and 0.1 mg/mL kanamycin sulfate (ThermoFisher Scientific) at 37 ℃ in 95% air and 5% CO_2_. Human-BM-MSCs, human adipose tissue-derived stem cells (h-ADSCs), and normal human dermal fibroblasts (NHDFs) were purchased from Lonza (Basel, Switzerland; h-BM-MSCs: PT-2501, h-ADSC: PT-5006, NHDF: CC-2511). Cells from passages 7 through 10 were used for Muse cell isolation.

Muse cells were isolated by fluorescence-activated cell sorting (FACS; BD FACS Aria^™^ II cell sorter, BD Biosciences [BD], San Jose, CA, USA). To isolate h-UC-SSEA-3 (+) cells, h-UC-MSCs were incubated with anti-SSEA-3 antibody (1:200 catalog #MA1-020; ThermoFisher Scientific) and stained with secondary antibody to fluorescein isothiocyanate (FITC)-conjugated anti-rat IgM (1:100, #112-095-075; Jackson ImmunoResearch Laboratories, West Grove, PA, USA).

To collect Muse cells, h-BM-MSCs, h-ADSCs, and NHDFs were incubated with anti-SSEA-3 antibody (1:1000, #330,302; BioLegend, San Diego, CA, USA) and stained with secondary antibody FITC-conjugated anti-rat IgM (1:100) as previously described [27]. Human-UC-, h-BM-, h-AT-, and h-dermal tissue (h-DT)-Muse cells were then collected as SSEA-3(+) fractions by FACS [1].

Muse cells and non-Muse cells stably expressing either green fluorescent protein (GFP), mCherry, or Akaluciferase (Akaluc) were prepared as described previously [28]. Akaluc/pcDNA3, provided by Dr. Satoshi Iwano [29], was inserted into a vector to construct the pWPXL vector. In brief, either pWPXL-GFP for lentivirus-GFP, pWPXL-mCherry for lentivirus-mCherry, or pWPXL-Akaluc for lentivirus-Akaluc were mixed with pMD2G and pCMV deltaR8.74 and transfected into LentiX-293 T packaging cells (Takara Bio, Shiga, Japan) using Lipofectamine 2000 (ThermoFisher Scientific). After 3 days, the viral supernatant was collected, centrifuged, and filtered through a 0.45-μm filter. To collect GFP- and Akaluc-labeled h-UC-SSEA-3(+) cells, h-UC-MSCs were infected with lentivirus, and then SSEA-3(+) cells were separated by FACS using allophycocyanin (APC)-conjugated goat anti-rat IgM (1:100) as the secondary antibody.

### Immunohistochemistry

To detect SSEA-3(+) cells in the h-UC, the tissue was fixed with 4% paraformaldehyde (PFA) in 0.1 M phosphate buffer (PB) overnight, embedded in paraffin, and cut into 3-μm thick sections. After deparaffinization and rehydration, sections were washed in phosphate-buffered saline (PBS), treated with 0.3% hydrogen peroxide (Fujifilm) in methanol (Fujifilm) for 30 min to inactivate the intrinsic peroxidase activity, and then incubated with 20% Block-Ace (KAC Co.; Kyoto, Japan)/5% bovine serum albumin (BSA, Nacalai Tesque, Kyoto, Japan)/0.3% Triton X-100 Fujifilm)/PBS solution for blocking. The sections were incubated overnight at 4 ℃ with anti-SSEA-3 antibody (1:200, #MA1-020; ThermoFisher Scientific) diluted with 5% Block-Ace/1% BSA/0.3% Triton X-100/PBS solution. After washing with PBS, the sections were incubated with horseradish peroxidase (HRP)-conjugated goat anti-rat IgM antibody (1:200). Expression of SSEA-3 was visualized by 3,3′-diaminobenzidine tetrahydrochloride (Fujifilm) and counterstained with Mayer’s hematoxylin (Fujifilm).

To evaluate the differentiation of h-UC-SSEA-3(+) cells and h-BM-Muse cells in mouse injured liver, mice anesthetized with isoflurane (Fujifilm) were transcardially perfused and fixed with 4% PFA in 0.1 M PB. The liver was dissected out, postfixed with 4% PFA in 0.1 M PB for 24 h, embedded in Optimal Cutting Temperature (OCT) compound (Sakura FineTechnical Co., Ltd., Tokyo, Japan), and then cut into 6-µm-thick frozen sections. STEM121 was used as a human cytoplasmic-specific marker to detect human-derived cells in the mouse liver. Albumin was used as a hepatocyte marker. Lymphatic vessel endothelial hyaluronan receptor 1 (Lyve-1) was as a liver sinusoidal endothelial cell marker. After washing with PBS, frozen sections were incubated with 20% Block-Ace/5% BSA/0.3% Triton X-100/PBS solution, and then incubated with mouse monoclonal antibody STEM121 (1:100, #Y40410; TaKaRa Bio Inc) followed by secondary antibody with Alexa Fluor 488-conjugated donkey anti-mouse IgG antibody (1:200, #715-546-150; Jackson ImmunoResearch). After washing with PBS, frozen sections were first incubated with goat anti-albumin (1:100, #A80-229A; Bethyl Laboratories, Montgomery, TX, USA) or rabbit anti-Lyve-1 antibodies (1:100, #NB600-1008; Novus Biologicals, Centennial, CO, USA), then with the secondary antibody. The primary and secondary antibodies used in this experiment are listed in Supplemental Table 1. The sections were counterstained with 4′,6-diamidino-2-phenylindole (DAPI; 1:500, D1306; ThermoFisher Scientific). Images were acquired by confocal laser microscopy (A1; Nikon). Ten randomly obtained images in each section were processed by ImageJ.1.53t to obtain a positive ratio for the albumin and Lyve-1 in STEM121(+) cells.

### Quantitative reverse transcriptase- polymerase chain reaction (qPCR)

Total RNA was collected using the NucleoSpin^®^ RNA XS (Macherey–Nagel, Duren, Germany), and cDNA was synthesized using Oligo(dT) 20 primer (ThermoFisher Scientific) and SuperScript^®^ III reverse transcriptase (ThermoFisher Scientific). DNA was amplified with the Applied Biosystems 7500 Fast real-time PCR system according to the manufacturer’s instructions. Data were processed using the ΔΔCT method [30]. Pluripotent markers were evaluated using a SYBR Green expression assay with the following primers: Nanog-F: 5′-CAGCTCGCAGACCTACATGA-3′, Nanog-R: 5′-CTCGGACTTGACCACCGAAC-3′; POU5F1-F: 5′-AACCCACACTGCAGCAGATCA-3′, POU5F1-R: 5′-ACACTCGGACCACATCCTTC-3′; SOX2-F: 5′-TCCAACATCCTGAACCTCAGC-3′, SOX2-R: 5′-TCTGCGTCACACCATTGCT-3′; ACTB-F: 5′-CATGTACGTTGCTATCCAGGC-3′, and ACTB-R: 5′-CTCCTTAATGTCACGCACGAT-3′. Trophectoderm markers were evaluated using TaqMan gene expression assay with the following primers: caudal type homeobox 2 (CDX2; Hs01078080_m1), inhibitor of DNA binding 2 (ID2; Hs04187239_m1), tumor protein p63 (TP63; Hs00978343_m1), glial cells missing-1 (GCM1; Hs00961601_m1), endogenous retrovirus group FRD member 1 (ERVFRD-1; Hs01652148_m1), and placental growth factor (PLGF; Hs00182176_m1). Germline-lineage markers were evaluated with the following primers: B lymphocyte-induced maturation protein 1 (BLIMP1; Hs00153357_m1), nanos C2HC-type zinc finger 3 (NANOS3; Hs00928455_s1), developmental pluripotency associated 3 (DPPA3; Hs01931905_g1), and SSEA-1 (Hs01106466_s1). Hematopoietic lineage markers were evaluated with the following primers: fetal liver kinase 1 (FLK-1; Hs00911700_m1), TIE2 (Hs00945149_m1), CD31 (Hs00169777_m1), c-KIT (Hs00174029_m1), CD34 (Hs02576480_m1), and protein tyrosine phosphatase receptor type C (PTPRC; Hs04189704_m1). ACTB (Hs99999903_m1) was used as an endogenous internal control.

### Surface marker expression

The antibodies used for double-staining with SSEA-3 were APC-labeled mouse anti-human HLA-ABC (1:100, #311409; BioLegend) and HLA-DR (1:100, #307609; BioLegend), phycoerythrin (PE)-labeled mouse anti-human CD29 (1:100, #556,049; BD), CD90 (1:100, #555596; BD), CD44 (1:100, #550989; BD), CD73 (1:100, #550257; BD), CD166 (1:100, #559263; BD), CD34 (1:100, #555822; BD), CD45 (1:100, #555483; BD), and CD271 (1:100, #557196; BD). Purified mouse anti-human CD105 (1:100, #555690; BD) and von Willebrand factor (vWF, 1:100, #555849; BD) antibodies were detected with Pacific Blue-labeled goat anti-mouse IgG (1:100, #P31582; ThermoFisher Scientific). Purified mouse anti-human CD133 (1:100, #130-090-422; Miltenyi Biotec), SSEA-4 (1:100, #330401; BioLegend), and HLA-G (1:50, #557577; BD) primary antibodies were detected by APC-labeled goat anti-mouse IgG (1:100, #115-136-146; Jackson ImmunoResearch Laboratories). These cells were analyzed by FACS Aria^™^ II (BD).

### Immunocytochemistry

Immunocytochemistry was performed as previously described [1]. Single-cell suspension-derived clusters generated from h-UC-SSEA-3(+) cells were fixed with 4% PFA in 0.1 M PB, embedded in OCT compound, and then cut into 6-µm-thick frozen sections. Differentiated cells derived from the h-UC-SSEA-3(+) cell cluster were grown in gelatin-coated dishes. Cells were fixed with 4% PFA in 0.1 M PB. After washing with PBS, the samples were incubated with 20% Block-Ace/5% BSA/0.3% Triton X-100/PBS solution for blocking, primary antibodies diluted in 5% Block-Ace/1% BSA/0.3% Triton X-100/PBS solution overnight at 4℃, washed 3 times with PBS, and then incubated with 0.2% Triton X-100/PBS solution containing secondary immunofluorescent antibodies (1:200) for 1 h at room temperature. After washing with PBS, the samples were stained with DAPI (1:500). The primary and secondary antibodies used in this experiment are listed in Supplemental Tables 1 and 2. Images were acquired by a confocal laser microscope (A1; Nikon).

### Droplet digital telomere repeat amplification protocol

The telomerase extension reaction was measured by droplet digital telomere repeat amplification protocol (ddTRAP) [31]. Whole-cell lysates were prepared in NP-40 lysis buffer (10 mM Tris–HCl [pH 8.0], 1 mM MgCl_2_, 1 mM EDTA, 1% [vol/vol] NP-40, 25 mM sodium deoxycholate, 10% [vol/vol] glycerol, 150 mM NaCl, 5 mM β-mercaptoethanol, and 0.1 mM AEBSF [4-(2-aminoethyl)benzenesulfonyl fluoride hydrochloride]), and added to extension reaction containing 1 × TRAP reaction buffer (10 × concentration: 200 mM Tris–HCl [pH 8.3], 15 mM MgCl_2_), 0.4 mg/mL BSA, 200 nM telomerase substrate primer (TS primer; 5′-AATCCGTCGAGCAGAGTT), and deoxynucleotide triphosphates (2.5 mM each), and incubated for 40 min at 25 ℃ followed by telomerase inactivation at 95 ℃ for 5 min, and then held at 4 ℃. The ddPCR reaction contained 1 × EvaGreen ddPCR Supermix (Bio-Rad, Hercules, CA, USA), 50 nM TS primer, 50 nM ACX primer, and an extension product. Droplets were produced in the QX200 droplet generator (Bio-Rad). PCR was performed on a C1000 thermocycler (Bio-Rad) with a ramp rate of 2.5 ℃/s between all steps. Activation of Taq polymerase (95 ℃ for 5 min) was followed by 40 cycles of 95 ℃ for 30 s, 54 ℃ for 30 s, and 72 ℃ for 30 s, then held at 12 ℃. Following PCR, fluorescence was read on the QX200 droplet reader (Bio-Rad). Data were analyzed using QuantaSoft software. The threshold between positive and negative droplets was determined by including controls such as a no-template lysis buffer control. HeLa cells were used as a positive control.

### Preparation of mouse embryonic stem cells

Mouse ESCs (TT2 cells) were cultured at 37 ℃ in Dulbecco’s modified Eagle’s medium (DMEM, ThermoFisher Scientific) containing 15% FBS, 0.1 mg/mL kanamycin sulfate, 0.1 mM MEM non-essential amino acid solution (ThermoFisher Scientific), 1 mM sodium pyruvate solution (ThermoFisher Scientific), 1000 U/mL leukemia inhibitory factor (LIF, MilliporeSigma), and 100 mM 2-mercaptoethanol (Nacalai Tesque) on mitomycin-C (Nacalai Tesque)-treated mouse embryonic fibroblast (MEF) feeder cells established from 12.5-day embryos of C57BL/6 mice, as previously described [3].

### Test for teratoma formation in immune-deficient mice testes

Human-UC-SSEA-3(+) cells (1 × 10^5^ cells) were suspended in PBS and injected into the testes of 8-week-old SCID mice (n = 6; Charles River Laboratories Japan, Yokohama, Japan) using a glass micropipette under anesthesia with isoflurane. Mice were killed by a lethal dose of isoflurane anesthesia at 6 months after injection. For controls, PBS (n = 2) was injected into the testes as a negative control and 5 × 10^5^ mouse ESCs (n = 4) were injected as a positive control; all were killed at 12 weeks after injection. Tissues were fixed with 4% PFA in 0.1 M PB, and 3-µm-thick paraffin sections were analyzed by hematoxylin–eosin staining.

### Migration assay

Human-UC-SSEA-3(+) cells and h-BM-Muse cells were prepared by cell sorting as described above. A Matrigel invasion chamber (BD) was used and the experiment was performed according to the manufacturer’s protocol. Serum-free medium alone or liver tissues from normal C57BL/6 mice in α-MEM were placed in the lower chamber. Human-UC-SSEA-3 (+) or h-BM-Muse cells (2.5 × 10^4^) were placed into the upper chamber and incubated at 37 ℃ in 5% CO_2_ for 24 h. Migrated cells were fixed with 4% PFA in 0.1 M PB for 15 min and stained with Mayer’s hematoxylin (Fujifilm). The number of migrated cells in each sample was counted under a 20 × objective in 4 fields, and the mean of 3 samples was calculated using ImageJ.1.53t.

### Preparation of dead cell fragments

Mouse-hepatic cell line Hepa1-6 (m-Hepa1-6) cells were infected with mCherry lentivirus and then expanded. To generate apoptotic-m-Hepa1-6, 2 × 10^6^ cells were treated with 100 μM rotenone (MilliporeSigma) and 100 μM antimycin A (MilliporeSigma) in DMEM for 24 h [9]. More than 95% of cells died and floated in the culture medium. The dead cell fragments were vortexed, filtered with a 40-µm cell strainer (SPL, Life Sciences, Pocheon, Korea), collected by centrifugation at 800 × *g* for 5 min, and stored in CELLBANKER 1 Plus (Juji-Field, Tokyo, Japan) at − 80 ℃ until use.

### In vitro live image observations of phagocytosis

Incubation of GFP-h-UC-SSEA-3(+) cells and apopototic-mCherry-m-Hepa1-6 dead cell fragments was recorded under a laser confocal microscope (Nikon) at 37 ℃ in 5% CO_2_ for live image analysis. The cells were counterstained with Hoechst 33342 (ThermoFisher Scientific). Images were captured every 15 min with a Z-series for three-dimensional imaging using a confocal laser microscope (A1; Nikon). Bitplane Imaris 9.7.2 software (Oxford Instruments, Abingdon, UK) was used to analyze the live image data.

### Next-generation sequencing

Total RNA from h-UC-SSEA-3(+) cells and h-BM-Muse cells was isolated by using the NucleoSpin RNA. The extracted RNA’s purity and concentration were evaluated using an Agilent 2100 bioanalyzer (Agilent Technologies, Palo Alto, CA, USA). The library was constructed by NEBNext Poly(A) mRNA Magnetic Isolation Module (New England Biolabs [NEB], Hitchin, UK) and NEBNext Ultra RNA Library Prep Kit for Illumina (NEB). Sequencing was performed on a NovaSeq 6000 (Illumina) by Rhelixa (Tokyo, Japan). Quality control and adapter trimming of sequencing data were performed using fastp version 0.20.0 [32]. DNA reads were mapped to the human reference genome DNA (build GRCh38.94) by HISAT2 version 2.2.1 with the default setting [33]. Gene counts from the mapped HISAT2 output and gene was generated by featureCounts version 2.0.128 for downstream expression analysis. Read counts for each gene were normalized to transcripts per million.

### In vivo dynamics of intravenously injected cells in mouse acute liver damage model

C57BL/6 mice (8–10 weeks old, Japan SLC, Shizuoka, Japan) were habituated under a specific pathogen-free environment at 24 ± 2 ℃ with a 12-h light–dark cycle at least for 1 week and allowed free access to standard institutional food and tap water except during injection. Mice received intraperitoneal injection of 5.0 mL/kg of carbon tetrachloride (CCl_4_; Fujifilm, Osaka, Japan) on day 0 and day 4. The CCl_4_ was dissolved in olive oil (Fujifilm) at 1:10. Human-UC-SSEA-3(+) cells (5 × 10^4^ cells) and h-BM-Muse cells (5 × 10^4^ cells) were injected into the tail vein of C57BL/6 mice 24 h after the second CCl_4_ intraperitoneal injection. At 7 days after the cell injection, the mice administered 1 mg AkaLumine-HCl (30 mM, Fujifilm) in distilled water. After 10 min, mice were killed by a lethal dose of isoflurane anesthesia. Each organ was removed, immersed in 500 µM AkaLumine-HCl in normal saline, and evaluated using an IVIS SpectrumCT in vivo imaging system (Perkin Elmer, Waltham, MA, USA). Living Image 4.5 software (Perkin Elmer) was used to analyze the regions of interest. The data are presented as the total photon flux (photons/s).

### Fluorescence in situ hybridization for X-inactive specific transcript analysis

The ubiquitously transcribed tetratricopeptide repeat gene on the X chromosome (UTX, known as the X chromosome-specific gene) and X-inactive specific transcript (XIST)-specific probes were used for fluorescence in situ hybridization analysis according to the manufacturer’s instructions (Chromosome Science Labo Inc., Sapporo, Japan). Cells were fixed with 4% PFA in 0.1 M PB, treated with 0.005% pepsin (Fujifilm)/0.1 N HCl at 37 ℃ for 3 min. After washing with PBS, cells were fixed with 4% PFA in 0.1 M PB, and then incubated with UTX-specific and XIST-specific probes overnight at 37 ℃. The cells were washed with 2 × saline sodium citrate (SSC) for 5 min at 37 ℃ with preheated 50% formamide/2xSSC for 20 min at 37 ℃ and with 1xSSC for 15 min at room temperature. Samples were counterstained with DAPI (1:500). Images were acquired with a confocal laser microscope (A1; Nikon) and the percent of XIST(+) cells was measured.

### Single-cell RNA sequencing (scRNA-seq)

Single-cell capture and cDNA synthesis were performed based on the TAS-seq protocol using the Rhapsody Single-Cell Analysis System (BD) [34]. All libraries were sequenced using Novaseq 6000 (Illumina, San Diego, CA, USA). Adapter trimming of sequencing data was performed using cutadapt version 2.10 [35]. Filtered cell barcode reads were annotated based on the Python script provided by BD. cDNA reads were mapped to reference RNA (build GRCh38 release-101) using bowtie2 version 2.4.2 [36]. The resulting count data were converted to a genes x cells matrix file. The sequencing and sequencing data processing was performed by ImmunoGeneTeqs (Tokyo, Japan).

Seurat R package version 3.2.2 in R version 4.0.2 was used for filtering, data integration, normalization, dimension reduction, and differentially expressed gene detection [37]. Genes detected in fewer than 3 cells were removed at the filtering step. Cells outside the thresholds of > 5000 expressed genes and 1–10% mitochondrial genes were considered low-quality cells and excluded. Our data were integrated with human embryo data: preimplantation stage (E-MTAB-3929) [38], pregastrulation stage (GSE136447) [38], and gastrulation stage (E-MTAB-9388) [39]. Data integration was performed by Seurat’s “FindIntegrationAnchors” function with the parameter “dims = 30”. After data integration, Seurat’s “ScaleData” function performed normalization. Clusters were graphically displayed by uniform manifold approximation and projection (UMAP) plot [40]. Cell clustering was performed by Seurat's “FindNeighbors” and “FindClusters” functions with the parameter “dims = 24, resolution = 0.8”. To evaluate the similarity for each cell type, Pearson’s product-moment correlation coefficient was calculated among all cell types using all genes by the “cor” function. We used Seurat’s “FindMarkers” function with the MAST algorithm to detect upregulated or downregulated genes of each type of the h-UC-Muse cells and h-embryo cells compared to the remaining cells [41]. Commonly upregulated or downregulated genes in each cell type (cluster 1, 3, 7 in h-UC-Muse cells, as well as epiblast, trophectoderm, primitive endoderm, primitive streak, and advanced mesoderm with a high correlation coefficient with them) were applied to Metascape (http://metascape.org), respectively [42]. Metascape was used for the pathway and process enrichment analysis with default parameters to explore the common biologic processes of h-UC-Muse cells and h-embryo cells [42]. For marker genes of each lineage, we selected genes annotated with Gene Ontology (GO) terms related to lineage development, such as “nervous system development”, “heart development”, and “liver development”. Dot plots and bar plots were generated by ggplot2 R package (https://ggplot2.tidyverse.org.), a heatmap by ComplexHeatmap R package [43, 44], and violin plots by Seurat’s “Vlnplot” function.

### Enzymatic methyl sequencing

All libraries were prepared using NEBNext Enzymatic Methyl-seq Kit (#E7120; NEB, Ipswich, MA, USA) and converted to libraries for DNBSEQ using MGIEasy Universal Library Conversion Kit (App-A, MGI Tech, Shenzhen, China) and AMPure XP beads (Beckman Coulter, Brea, CA, USA). To monitor the C to T conversion rate, unmethylated lambda DNA was added to the genomic DNA samples before library construction. The libraries were sequenced using DNBSEQ-G400RS (MGI Tech). Adapter trimming of sequencing data was performed using fastp version 0.20.0 [32]. DNA reads were mapped to reference human genome DNA (build hg19.p13), duplicate reads were removed and the methylation call for every single C analyzed was removed, and then the number of methylated and unmethylated reads was counted using bismark version v0.23.1 [45], Bowtie2 version 2.4.5 [46], and samtools version 1.7 [47]. For the total unique CpG, we calculated the number of genomic CpG sites where DNA methylation was detected. Methylation was defined as detected if the count was 1 (1x), 5 (5x), or 10 (10x) or higher, respectively. Regarding mean coverage, we extracted regions of the genome where the depth was 1 (1x), 5 (5x), or 10 (10x) or higher, respectively, and calculated the mean depth for each category. The lambda DNA genome was rebuilt as an extra reference for calculating the C to T conversion rate in each sample. Sequencing was performed by Kazusa DNA Research Institute (Chiba, Japan).

Methylkit R package version 1.18.0 [48] and genomation version 1.24.0 [49] in R version 4.1.1 were used for filtering, determining the methylation level, calculating correlations, and identifying the differentially methylated regions. Our data were integrated with human embryo data (GSE49828) [50]. Genes detected with a coverage of less than 10 reads were removed at the filtering step. To analyze the methylation level of each CpG site, the following algorithm was applied: number of methylated C divided by the total number of methylated C and unmethylated T at the exact positions of the reference genome was calculated as the DNA methylation level of the CpG site. Every CpG site with a read depth ≥ 1 was summed and counted as the total CpG coverage of the sample. For quantifying the DNA methylation level in each sample, only the CpG sites with a coverage of more than 10 reads were considered and subjected to the 100-base pair (bp)-tile-based DNA methylation algorithm [51]. First, the genome was binned into consecutive 100-bp tiles. The number of methylated C divided by the total number of methylated C and unmethylated T captured in the 100-bp tile, was interpreted as the 100-bp tile averaged DNA methylation level. The CpG density of every CpG site was calculated as the total number of all CpG dinucleotides located within 50 bp upstream and 50 bp downstream of this CpG site. In contrast, the CpG density of every 100-bp tile was then calculated as the average CpG density of all CpG sites in this 100-bp tile. Using these data, we generated a correlation matrix, a heatmap with hierarchical clustering, and a principal component analysis (PCA) plot of DNA methylation between human embryos and Muse cells. To explore the common GO terms of embryonic cells and UC-Muse cells in low methylated genes, genes with low methylation (< 10%) were extracted from h-UC-Muse cells with the same level of methylation as h-post-implantation blastocysts (< 1% variation). Genes with a methylation status that varied by more than 30% or less than − 30% in the CpG region around 1000 bp from the transcription start point in h-UC-Muse cells compared to h-BM-, h-AT-, and h-DT-Muse cells, respectively, were extracted. Metascape was used for the pathway and process enrichment analysis with default parameters [42]. For marker genes of each lineage, genes annotated with GO terms related to lineage development, such as triploblastic, extraembryonic-lineage, germline-lineage, and hematopoietic-lineage differentiation, were selected.

### In vitro differentiation of Muse cells

In vitro differentiation of Muse cells into extraembryonic-lineage marker (+) cells was conducted according to the previously described method with minor modifications [52]. For extraembryonic-lineage induction, h-UC-, h-BM-, h-AT-, and h-DT-Muse cells (1 × 10^4^ cells/cm^2^) were cultured in DMEM supplemented with 2% FBS, 10 ng/mL bone morphogenic protein‐4 (BMP4, Fujifilm), 20 µM SB431542 (Fujifilm), and 20 µM SU5402 (Fujifilm) on adherent culture for 3 weeks.

In vitro differentiation into germline-lineage marker positive cells was conducted according to the previously described method with minor modifications [53]. Human-UC-, h-BM-, h-AT-, and h-DT-Muse cells (1 × 10^4^ cells/cm^2^) were cultured in DMEM supplemented with 10% FBS, 50 ng/mL Activin A Fujifilm), 3 µM CHIR99021 (Fujifilm), and 10 µM Y27632 (Fujifilm) on adherent culture for 2 days (incipient mesoderm-like cell induction) [53]. After 2 days of incipient mesoderm-like cell induction, cells were cultured in DMEM supplemented with 10% FBS, 200 ng/mL BMP4, 1000 U/mL LIF (MilliporeSigma), 100 ng/mL stem cell factor (SCF, Fujifilm), 50 ng/mL epidermal growth factor (EGF, Fujifilm), and 10 µM Y27632 (Fujifilm) in suspension culture using low-cell binding V-bottom 96-well plates for 4 days.

In vitro differentiation into hematopoietic-lineage marker (+) cells was conducted according to the previously described method with minor modifications [54]. Human-UC-, h-BM-, h-AT-, and h-DT-Muse cells (1 × 10^4^ cells/cm^2^) were cultured in DMEM supplemented with 5% FBS and 100 ng/mL BMP4 on adherent culture for 2 days and then in DMEM supplemented with 5% FBS and 20 ng/mL BMP4 on adherent culture for 2 days. After 4 days of adherent culture, the cells were cultured in DMEM supplemented with 5% FBS, 40 ng/mL vascular endothelial growth factor (Fujifilm), 50 ng/mL SCF for 2 days and then in DMEM supplemented with 5% FBS, 50 ng/mL SCF, 10 ng/mL thrombopoietin (Fujifilm), 50 ng/mL interleukin-3 (Fujifilm), 50 ng/mL granulocyte colony stimulating factor (Fujifilm) and 50 ng/mL FMS-like tyrosine kinase 3 ligand (Fujifilm) in suspension culture using low-cell binding V-bottom 96-well plates for 11 days.

### Western blotting

Cells were washed with cold PBS twice and lysed by RIPA Lysis and Extraction Buffer (ThermoFisher Scientific) supplemented with a protease inhibitor (ThermoFisher Scientific) and a phosphatase inhibitor (MilliporeSigma). Cell lysates were centrifuged at 11,000 × *g* at 4 ℃. The supernatant was transferred to new tubes, and protein amounts were measured using a bicinchoninic acid protein assay kit (ThermoFisher Scientific) according to the manufacturer’s instructions. Sodium dodecyl sulfate–polyacrylamide gel electrophoresis (SDS-PAGE) was performed using SDS-PAGE gels, and the proteins were then transferred to polyvinylidene difluoride membranes (MilliporeSigma). Membranes were blocked using 2% BSA/Tris-buffered saline with 0.05% Tween-20 (TBST), then incubated overnight at 4 ℃ in primary antibodies diluted in 2% BSA/TBST. After 3 washes with TBST, the membranes were incubated with secondary antibodies (diluted 1:5000 in 2% BSA/TBST for 1 h at room temperature. After washing with TBST, the blots were developed by Pierce ECL Plus Western Blotting Substrate (ThermoFisher Scientific). Images were acquired using Fusion FX imaging systems (Vilber). Band intensity was quantified by ImageJ software. The primary and secondary antibodies used in this experiment are listed in Supplemental Tables 1 and 2.

### Statistics

The data were analyzed by 1-way analysis of variance and an unpaired Student’s t-test to determine statistical significance using Microsoft^®^ Excel software. All data represent 3 independent experiments. The results are presented as the mean ± standard error of the mean. A p-value of less than 0.05 was considered significant.

## Results

### Characterization of h-UC-SSEA-3(+) cells

**Fig. 1:**
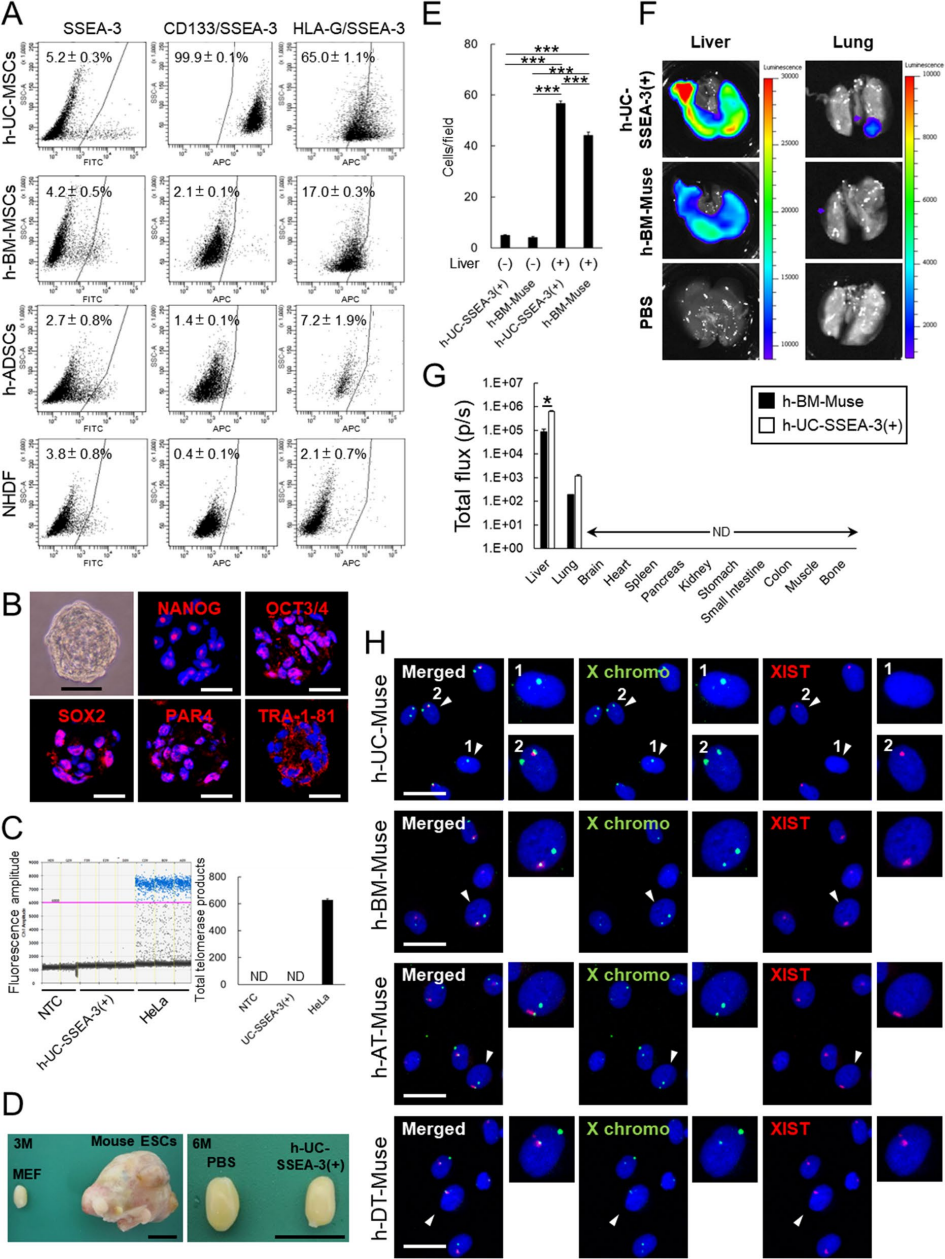
Characterization of SSEA-3(+) cells from h-UC-MSCs. **A** Expression of CD133 and HLA-G in h-UC-MSCs, h-BM-MSCs, h-ADSCs, and NHDF. **B** Clusters formed in single-cell suspension culture from h-UC-SSEA-3(+) cells. These clusters expressed Nanog, Oct3/4, Sox2, PAR4, Tra-1-81. **C** Telomerase activity in HeLa cells, h-UC-SSEA-3(+) cells, and non-template control (NTC) measured by ddPCR. Each dot on the ddPCR output represents a unique droplet that is either positive or negative for a fluorescent signal whose threshold was 6000. **D** SCID mouse testes injected with MEF (3 months), mouse ESCs (3 months), PBS (6 months), and h-UC-SSEA-3(+) cells (6 months). **E** In vitro migration of h-UC-SSEA-3(+) cells and h-BM-Muse cells toward damaged liver tissue slice. **F** In vivo dynamics of intravenously injected h-UC-SSEA-3(+) cells and h-BM-Muse cells in the acute liver injury model. One week after intravenous administration, the Akaluc signal was detected predominantly in the liver while it was stronger in the h-UC-Muse group than in the h-BM-Muse group. **G** Total flux by ex vivo imaging. In organs other than the liver and lung, the Akaluc signal was under the detection limit. **H** Expression of XIST in h-UC-, h-BM-, h-AT, and h-DT-Muse cells

The localization of SSEA-3(+) cells, a marker used for Muse cell isolation [1], in h-UC was examined by immunohistochemistry. Most SSEA-3(+) cells exhibiting a round shape and a 9.5 - 13.8 µm diameter were sparsely distributed in the Wharton's jelly loose connective tissue and not associated with specific histologic structures (Fig. S1A). To isolate SSEA-3(+) cells, the UC obtained from 5 donors (named h-UC1 ~ 5) was minced into ~ 5-mm^3^ pieces, and adherent cells were collected as h-UC-MSCs (Fig. S1B and Supplemental Table 3). SSEA-3(+) cells comprised 5.2 ± 0.3% of h-UC-MSCs (Figs. 1A, S2A and Supplemental Table 3). This percentage is comparable to or higher than that previously reported in h-BM-MSCs (4.2 ± 0.5%) [55, 56], h-ADSCs (2.7 ± 0.8%) [3, 57], and NHDFs (3.8 ± 0.8%) [58, 59] (Figs. 1A, S2B). The h-UC-MSCs from h-UC1 ~ 5 were then separated into SSEA-3(+) and (-) cells and the pluripotency gene expression level was examined. The expression of OCT3/4 was significantly higher in h-UC-SSEA-3(+) cells than in h-UC-SSEA-3(-) cells in all batches, while the expression of NANOG was significantly higher in h-UC-SSEA-3(+) cells from h-UC2, h-UC3, and h-UC5; and the expression of Sox2 was significantly higher in h-UC-SSEA-3(+) cells than in the corresponding h-UC-SSEA-3(-) cells (Fig. S3A). The following experiments used cells obtained from h-UC2, h-UC3, and h-UC5, which exhibited consistently higher expression of all 3 markers among the h-UC-SSEA-3(+) cells compared with the h-UC-SSEA-3(-) cells.

All of the h-UC-SSEA-3(+) cells were positive for MSC markers CD105, CD90, CD44, CD73, and CD166, while 61.6 ± 3.2% of them expressed CD29 (Fig. S2A). The expression of hematopoietic markers CD34 and CD45, the neural crest stem cell marker CD271, and the vascular endothelial cell marker vWF was under the detection limit (Fig. S2A). SSEA-4, a member of the globo series of glycosphingolipids to which SSEA-3 belongs, was expressed in 97.6 ± 0.2% of h-UC-SSEA-3(+) cells, similar to that in h-AT-Muse cells (94.0 ± 0.6%), but the expression was higher than that in h-BM-Muse cells (66.8 ± 0.6%) and h-DT-Muse cells (77.9 ± 0.6%; Fig. S2C). CD133, a marker for ESCs, HSCs, NSCs, and cancer stem cells, was expressed in 99.9 ± 0.1% of h-UC-SSEA-3(+) cells, a percentage substantially higher than that in h-BM-Muse cells (2.1 ± 0.1%), h-AT-Muse cells (1.4 ± 0.1%), and h-DT-Muse cells (0.4 ± 0.1%; Figs. 1A). Regarding HLA, HLA-ABC was detectable in 100% of h-UC-SSEA-3(+), whereas HLA-DR was under the detection limit (Fig. S2A). These results were similar to those in h-BM-Muse cells [10]. HLA-G was highly positive in h-UC-SSEA-3(+) cells (65.0% ± 1.1%) compared with h-BM-Muse cells (17.0 ± 0.3%), h-AT-Muse cells (7.2 ± 1.9%), and h-DT-Muse cells (2.1 ± 0.7%; Figs. 1A).

When h-UC-SSEA-3(+) cells were cultured in single-cell suspension culture, cell clusters resembling ESC-derived embryoid bodies that expressed Nanog, Oct3/4, Sox2, PAR4, and Tra-1-81 formed within 1 week (Figs. 1B, S3B). When these clusters were individually transferred onto gelatin-coated dishes and cultured without cytokine induction for 14 days, the adherent cells from the cluster proliferated and spontaneously expressed cytokeratin 7 (endodermal marker), smooth muscle actin (mesoderm marker), and neurofilament-M (ectodermal marker; Fig. S3C). These results were similar to those of h-BM-, h-AT-, and h-DT-Muse cells [1–3].

Telomerase activity evaluated by ddTRAP in HeLa cells was high while that in h-UC-SSEA-3(+) cells was under the detection limit (Fig. 1C). To examine tumorigenicity, mouse ESCs and h-UC-SSEA-3(+) cells (both at 1 × 10^5^ cells), were injected into the testes of SCID mice. Although ESCs formed teratomas within 3 months, no teratoma formation was observed in h-UC-SSEA-3(+) cells at 6 months (Figs. 1D, S3D). These results were similar to those of h-BM-, h-AT-Muse cells, and human amniotic membrane-SSEA-3(+) cells [1, 3, 6].

### Behavior of intravenously injected h-UC-Muse cells in a mouse acute liver damage model

In various animal disease models, intravenously administered Muse cells are reported to selectively migrate to sites of damage and repair tissues by differentiating into tissue-constituting cells after phagocytosing damaged/apoptotic cells [9–11, 55, 59, 60]. First, the migration ability of h-UC-SSEA-3(+) cells and h-BM-Muse cells was compared in vitro using a Matrigel invasion chamber. Serum-free medium alone or mouse liver tissue slices were placed in the lower chamber, h-UC-SSEA-3(+) cells or h-BM-Muse cells were seeded into the upper chamber, and the number of cells that migrated toward the lower chamber was counted at 24 h. No significant difference was detected between h-BM-Muse cells (4.0 ± 0.5 cells/field) and h-UC-SSEA-3(+) cells (4.9 ± 0.3 cells/field) in serum-free medium. When liver tissue was placed in the lower chamber, significantly more h-UC-SSEA-3(+) cells (56.7 ± 1.0 cells/field) migrated than h-BM-Muse cells (44.1 ± 1.3 cells/field; p < 0.001; Fig. 1E).

To evaluate the phagocytotic ability of h-UC-SSEA-3(+) cells in vitro, GFP-labeled h-UC-Muse cells were supplied with dead cell fragments collected from rotenone- and antimycin A-treated mCherry-labeled mouse hepatic cell line Hepa1-6 for 24 h. The mCherry signals located within GFP-h-UC-Muse cells were extracted by Imaris software. The mCherry(+) dead cell fragments taken up by GFP-h-UC-Muse cells were distributed in the cytoplasm and nucleus (Fig. S4A–C and Movie 1). When cells containing at least 1 mCherry(+) particle were counted as phagocytosis-positive, 92.6 ± 1.2% of GFP-h-UC-SSEA-3(+) cells were positive at 24 h. These results were similar to the findings in h-BM-Muse cells [9]. Furthermore, when the expression of phagocytosis receptor genes was investigated using next-generation sequencing data, h-UC-Muse cells expressed a significantly lower level of CD36 (p < 0.05) and a significantly higher level of ICAM1 (p < 0.05) than h-BM-Muse cells. In contrast, the expression levels of other receptors were similar between h-UC-Muse and h-BM-Muse cells (Fig. S4D).

To evaluate migration to the site of damage and differentiation into tissue components in vivo, 5 × 10^4^ h-UC-SSEA-3(+) cells or h-BM-Muse cells were intravenously injected into a mouse acute liver injury model induced by intraperitoneal injection of carbon tetrachloride (CCl_4_). At 1 week, the in vivo dynamics of the injected cells revealed a significantly higher accumulation of h-UC-SSEA-3(+) cells than h-BM-Muse cells in the liver (p < 0.05) and both h-UC-SSEA-3(+) and h-BM-Muse cells accumulated to the lung at similar levels but to a lesser extent than in the liver. Both cell types were under the detection limit in organs other than the liver and lung (Figs. 1F-G, S5A). In immunohistochemistry at 2 weeks, h-UC- and h-BM-Muse cells, identified as human cytoplasmic marker STEM121(+) cells, were present in the liver. Of the STEM121(+) cells, hepatocyte marker albumin(+) cells comprised 53.5 ± 8.6% and sinusoid endothelial marker Lyve-1(+) cells comprised 37.1 ± 6.6% in the liver of the h-UC-SSEA-3(+) group, comparable to the h-BM-Muse group (51.0 ± 2.1%, and 33.5 ± 8.7%, respectively; Fig. S5B–E). These results are similar to those of previous reports demonstrating that h-BM-Muse cells administered to liver injury models selectively accumulate in the liver and differentiate into liver components [60–62].

In summary, the above results suggest that the characteristics of h-UC-SSEA-3(+) cells were similar to those of h-adult tissue-Muse cells, namely h-BM-, h-AT-, and h-DT-Muse cells. Therefore, h-UC-SSEA-3(+) cells are referred to as ‘h-UC-Muse cells’ in the following text.

### XIST expression

Pluripotent stem cells (PSCs) are classified into naïve and primed PSCs on the basis of the X chromosome status and differentiation potential [63]. The naive state resembles the inner cell mass prior to implantation whose X chromosomes are both active, indicated by XIST-negativity. The prime state, on the other hand, resembles the post-implantation epiblast, in which either of the X chromosomes is inactivated via XIST, indicated by XIST-positivity. In h-UC-Muse cells, 81.8 ± 1.9% were XIST-positive and ~ 18% were XIST-negative. In h-BM-, h-AT-, and h-DT-Muse cells, all the cells were XIST-positive (Fig. 1H).

### scRNA-seq

After excluding low-quality cells such as doublets and dead cells, a total of 4577 single cells (1566 from h-UC-Muse cells [batch #h-UC3], 835 from h-BM-Muse cells, 1288 from h-AT-Muse cells, and 618 from h-DT-Muse cells) were used for analysis. To compare human Muse (h-Muse) cells and human embryo (h-embryo) cells, we referred to previously reported data comprising 3279 cells (1529 from h-embryo cells [E3-7, pre-implantation stage, E-MTAB-3929] [38], 555 from h-embryo cells [E8-14, pre-gastrulation, GSE136447] [38], 1195 from h-embryo cells [E16, Gastrulation, E-MTAB-9388]) [39].

**Fig. 2:**
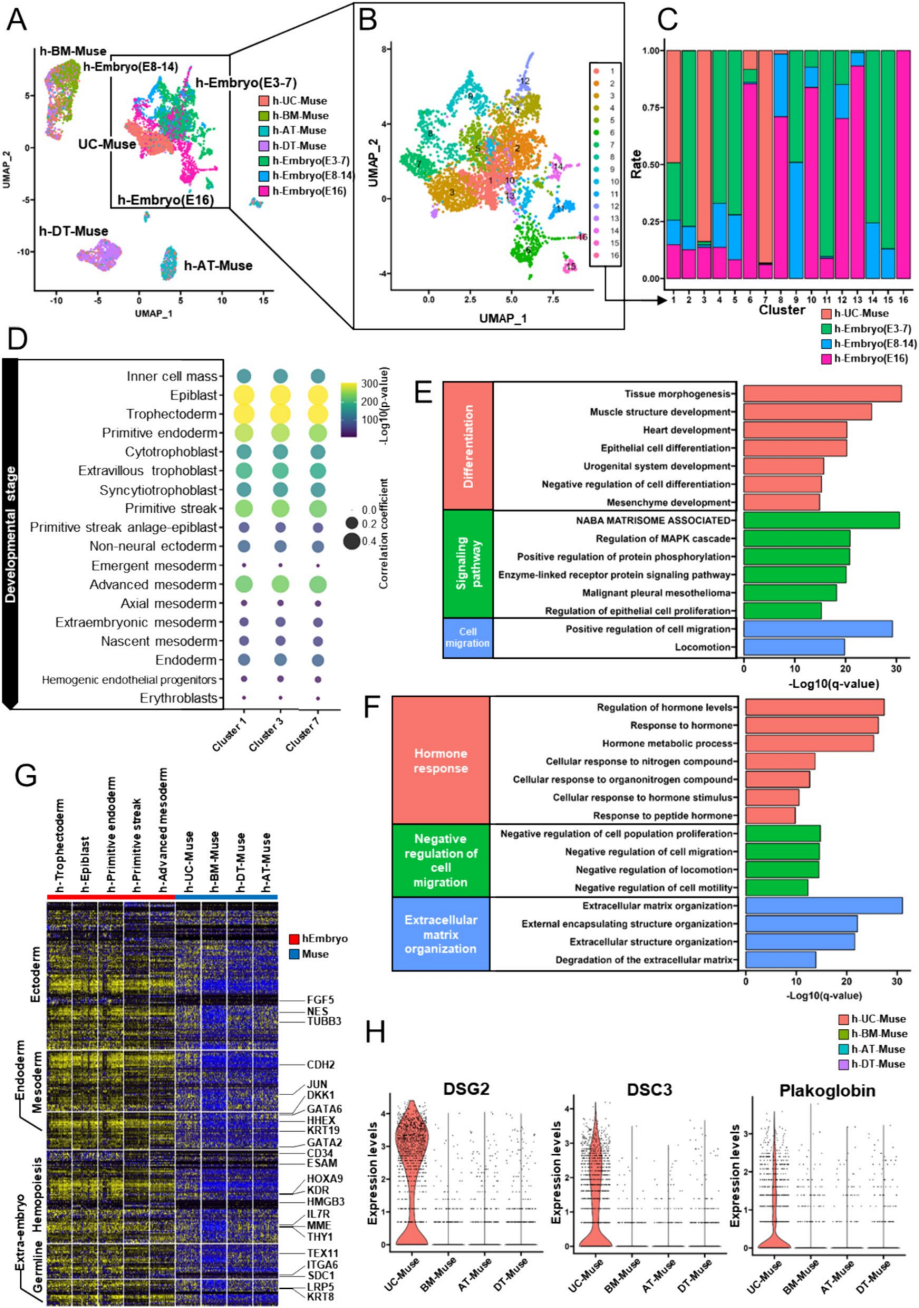
Comparison of human Muse cells and h-embryo cells in scRNA-seq. **A** UMAP of h-UC-, h-BM-, h-AT, h-DT-Muse cells, integrated with published datasets of h-embryo (E3-7) [38], h-embryo (E8-14) [38], and h-embryo (E16) cells [39]. **B** The enlarged image of UMAP of h-UC-Muse cells and h-embryo cells in the square of (**A**). **C** The ratio of h-UC-Muse cells, h-embryo (E3-7), h-embryo (E8-14), and h-embryo (E16) cells in clusters 1–16 classified in (**B**). **D** Dot plots of the correlation coefficient for cell types of h-embryos in clusters 1, 3, and 7 of h-UC-Muse cells. The -log10 (p-value) is indicated by the color intensity. The dot size indicates the correlation coefficient. **E** GO term of commonly upregulated genes in each cell type (clusters 1, 3, 7 in h-UC-Muse cells, as well as epiblast, trophectoderm, primitive endoderm, primitive streak, and advanced mesoderm with a high correlation coefficient among them) compared with the remaining h-embryo cells. **F** GO term of commonly downregulated genes in each cell type (clusters 1, 3, 7 in h-UC-Muse cells, as well as epiblast, trophectoderm, primitive endoderm, primitive streak, and advanced mesoderm with a high correlation coefficient among them) compared to the remaining h-embryo cells. **G** Heatmap of the lineage-specific marker genes in each type of h-embryo and h-Muse cell. **H** Violin plot showing the expression levels of DSG2, DSC3, and plakoglobin in h-UC-, h-BM-, h-AT, and h-DT-Muse cells

Muse cells and h-embryo cells were plotted as distinct clusters based on their gene expression using the UMAP dimensionality reduction technique. A different distribution was observed for each Muse cell type; h-UC-Muse cells were closer to h-embryo cells from E3-7, E8-14, and E16 compared with other types of Muse cells (Fig. 2A). We then focused on h-UC-Muse cells and h-embryo cells from E3-7, E8-14, and E16. These cells were divided into 16 clusters, in which most h-UC-Muse cells projected to clusters 1, 3, and 7 (Fig. 2B–C). We examined the similarity between h-UC-Muse cells and h-embryo cells and found that cluster 1 (human epiblast [h-epiblast]: 0.774 [correlation coefficient], human trophectoderm [h-trophectoderm]: 0.764), cluster 3 (h-epiblast: 0.768, h-trophectoderm: 0.760), and cluster 7 (h-epiblast: 0.754, h-trophectoderm: 0.741) in h-UC-Muse cells was highly correlated with h-trophectoderm (E3-7) and h-epiblast (E8-14) cells in the h-post-implantation blastocyst (Fig. 2D).

Pathway and process enrichment analysis was performed after extraction of commonly upregulated or downregulated genes in each cell type (clusters 1, 3, 7 in h-UC-Muse cells, as well as epiblast, trophectoderm, primitive endoderm, primitive streak, and advanced mesoderm with a high correlation coefficient among them) compared with the remaining h-embryo cells. Upregulated genes were categorized into differentiation, signaling pathway, and cell migration (Fig. 2E). On the other hand, downregulated genes were categorized into hormone response, negative regulation of cell migration, and extracellular matrix organization (Fig. 2F). Regarding genes related to ectodermal-, mesodermal-, endodermal-, hematopoietic-, germline- and extraembryonic cell-lineages, the gene expression pattern of h-UC-Muse cells resembled that of h-embryo cells, particularly h-trophectoderm, h-epiblast, h-primordial endoderm, h-primitive streak, and h-advanced mesoderm cells, rather than that of h-adult tissue-Muse cells (Fig. 2G).

Genes with a fold-change greater than 1.5 and adjusted p value less than 0.05 in h-UC-Muse cells compared to h-BM-Muse cells were extracted. The identified genes were common to any of 3 GO terms related to cell migration (cell migration and positive regulation of cell migration; Fig. S6A, B). Compared with h-BM-Muse cells, h-UC-Muse cells had higher expression levels of genes involved in cell migration, such as bradykinin receptor B1 (BDKRB1; bradykinin receptor produced during inflammation and tissue injury) [64], tissue factor 3 (F3, also known as CD142: involved in the migration of trophoblast cells) [65], coronin-1A (CORO1A; actin regulator) [66, 67], podocalyxin-like (PODXL; adhesion inhibitor) [68], and S1P receptor 1 (S1PR1: involved in the migration of immune cells, stem/progenitor cells and Muse cells) [10, 69, 70] (Fig. S6C). The expression of S1PR2 [10], which contributes to Muse cell migration, did not differ significantly between h-UC-Muse cells and h-BM-Muse cells (Fig. S6D).

Human-UC-Muse cells had higher expression levels of desmosome-related genes, such as desmoglein 2 (DSG2), desmocollin 3 (DSC3), and plakoglobin (PG), which are known to be expressed in early human embryos [71, 72], compared with the adult tissue-Muse cells (h-BM-, h-AT-, and h-DT-Muse cells; Fig. 2H).

### DNA methylation analysis

**Fig. 3:**
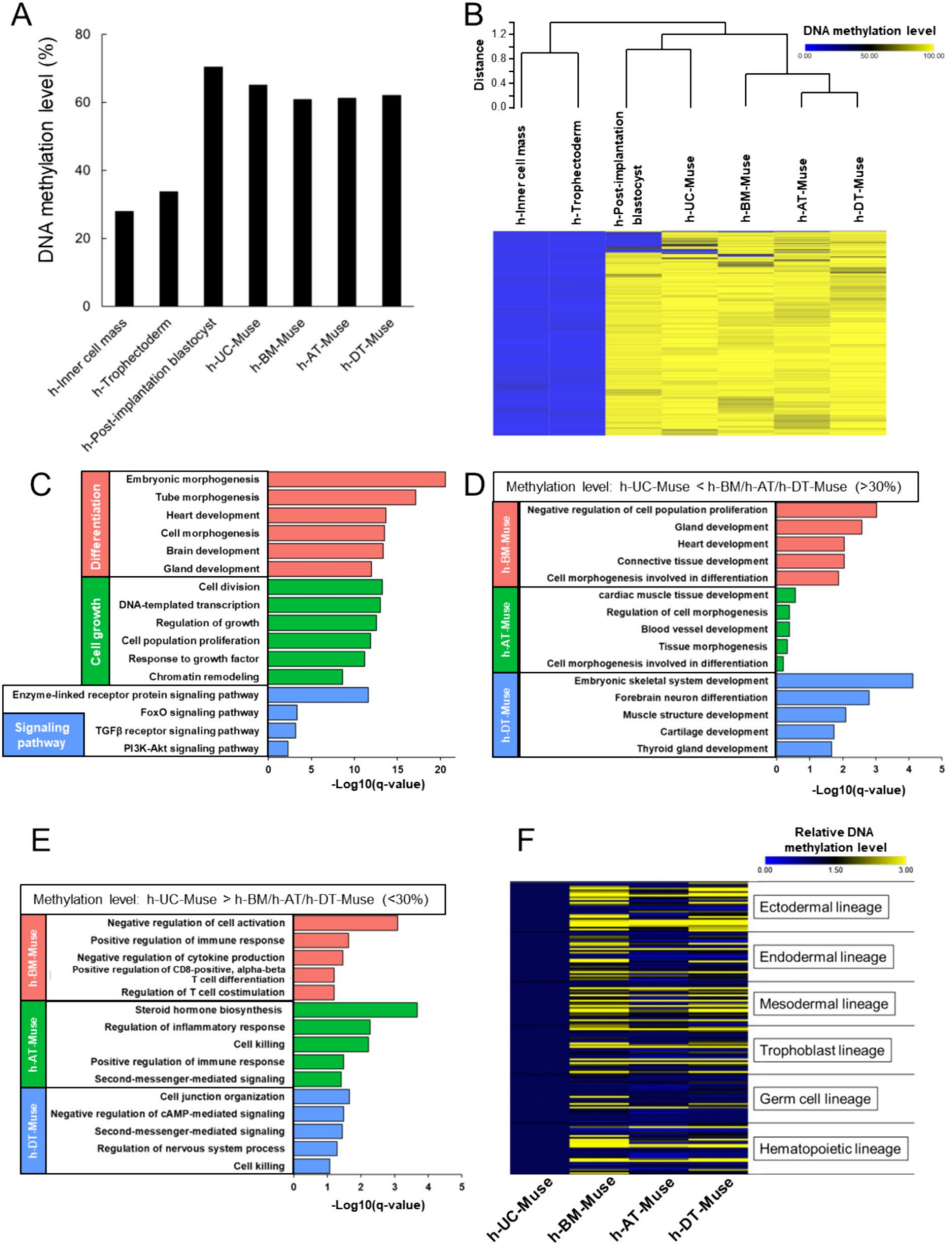
Comparison of h-Muse cells and h-embryo cells in enzymatic methyl sequencing. **A** Methylation landscape of h-Muse cells and h-embryo cells. The averaged DNA methylation levels were calculated based on the overlapped 100-bp tiles detected in h-Muse cells and h-embryo cells. **B** Heatmap with hierarchical clustering used a distance metric based on Pearson’s correlation coefficients and average linkage in h-Muse cells and h-embryo cells. **C** GO term of genes with low methylation levels (< 10%) in both h-UC-Muse cells and h-post-implantation blastocysts. **D** GO term of hypomethylated genes with relative methylation levels lower than 30% in h-UC-Muse cells compared with h-adult tissue-Muse cells. **E** GO term of hypermethylated genes with relative methylation levels higher than 30% in h-UC-Muse cells compared with h-adult tissue-Muse cells. **F** Heatmap of the lineage-specific marker genes in h-Muse cells. Values of h-UC-Muse cells were set as 1

To examine DNA methylation status in h-UC (batch #h-UC3)-, h-BM-, h-AT-, and h-DT-Muse cells, enzymatic methyl sequencing was conducted (Supplemental Table 4). For comparison, previously reported h-embryo data (GSE49828) were used (Supplemental Table 4) [50]. The methylation levels of 100-bp tiles in CpG and the methylation pattern were similar to each other among the 4 Muse cell types and also between h-post-implantation blastocysts and the 4 Muse cell types (Fig. 3A and Fig. S7A). In the heatmap with hierarchical clustering and the PCA plot, the DNA methylation pattern of h-UC-Muse cells was more similar to that in h-post-implantation blastocysts than that in h-adult tissue-Muse cells (Fig. 3B and Fig. S7B). We further focused on comparing h-UC-Muse cells and post-implantation blastocysts as scRNA-seq demonstrated similarity between h-UC-Muse cells and post-implantation blastocysts at several points. Pathway and process enrichment analysis showed that genes with low methylation levels (< 10%) in both h-UC-Muse cells and h-post-implantation blastocysts were involved in differentiation, cell growth, and signaling pathways (Fig. 3C).

The methylation status of h-UC-Muse cells was compared with that of h-adult tissue-Muse cells in the CpG region ± 1000 bp around the transcription start sites. Hypomethylated genes with relative methylation levels lower than 30% and hypermethylated genes with relative methylation levels higher than 30% in h-UC-Muse cells were compared to those in h-adult tissue-Muse cells by pathway and process enrichment analysis. Genes hypomethylated in h-UC-Muse cells than in h-adult tissue-Muse cells were involved in differentiation; h-BM-Muse cells: negative regulation of cell proliferation, gland development, and heart development; h-AT-Muse cells: cardiac muscle tissue development, regulation of cell morphogenesis, and blood vessel development; and h-DT-Muse cells: embryonic skeletal system development, forebrain neuron differentiation, and muscle structure development (Fig. 3D). Hypermethylated genes in h-UC-Muse cells compared with h-adult tissue-Muse cells were partially involved in immune response (h-BM-Muse cells: negative regulation of cell activation and positive regulation of immune response, h-AT-Muse cells: steroid hormone biosynthesis, regulation of inflammatory response, and cell killing; and h-DT-Muse cells: cell junction organization and negative regulation of cAMP-mediated signaling; Fig. 3E).

Furthermore, the methylation level of genes related to ectodermal-, mesodermal-, endodermal-, hematopoietic-, germline-, and extraembryonic cell-lineages was lower in h-UC-Muse cells than in h-adult-tissue-Muse cells (Fig. 3F).

### Extraembryonic-lineage marker expression by in vitro induction

In contrast to h-adult tissue-Muse cells, h-UC-Muse cells contained XIST(-) cells, a characteristic of naïve PSCs. Unlike primed PSCs, naïve PSCs differentiate into extraembryonic-lineages [63]. The 4 Muse cell types were treated for 3 weeks with cytokines and reagents known to differentiate ESCs into syncytiotrophoblast-lineage cells, with minor modifications [52].

**Fig. 4:**
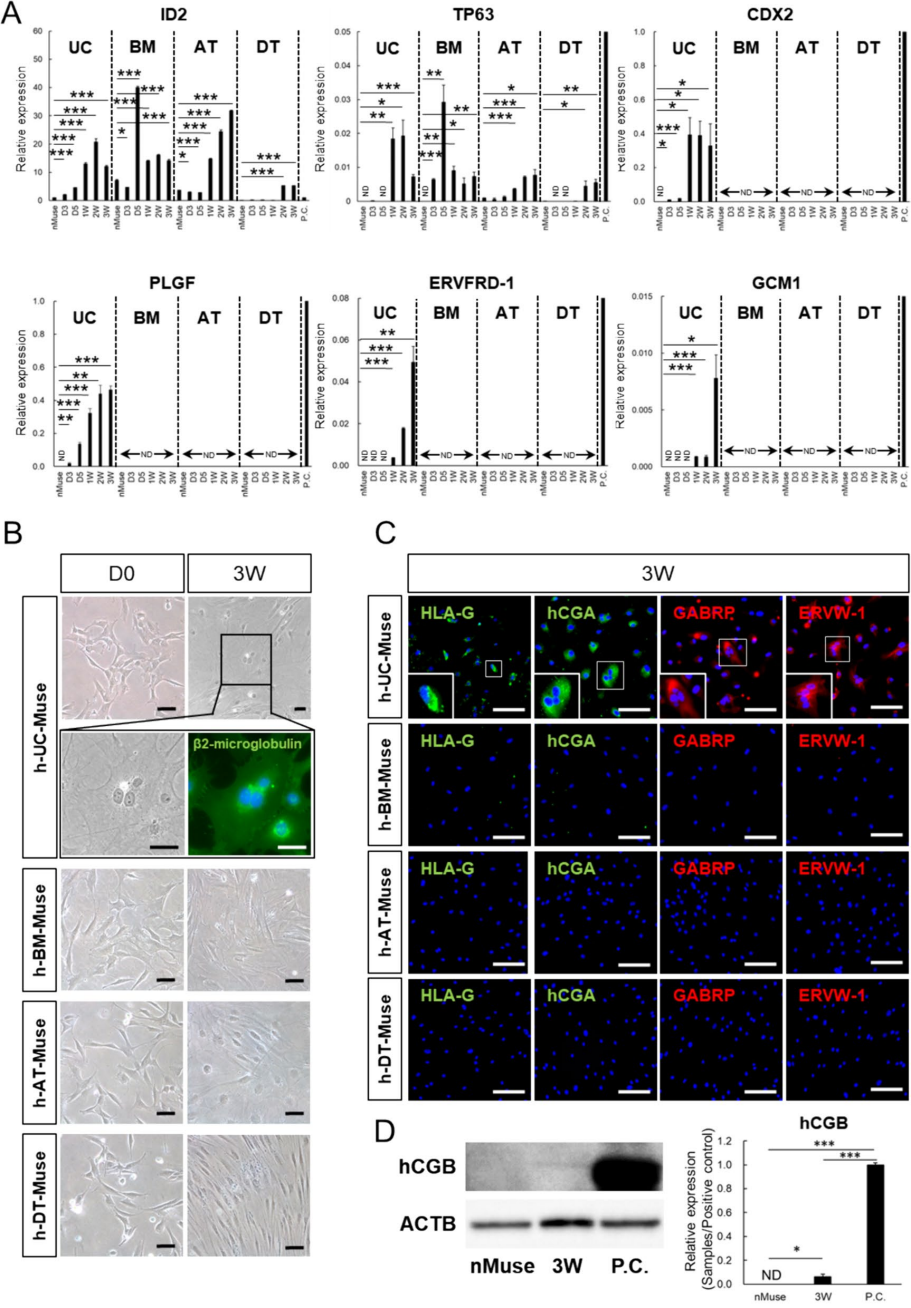
In vitro differentiation of h-UC-Muse cells into extraembryonic-lineage marker(+) cells. **A** Expression of extraembryonic cell-lineage genes in the 4 types of naïve Muse cells, as well as in those from day 3 to 3 weeks after induction (normalized by ACTB). JEG3 was set as a positive control (P.C.). Values of JEG3 were set as 1. **B** Extraembryonic-lineage induction of h-Muse cells. Human-UC-Muse cells at 3 weeks after induction contained multinucleated cells in phase contrast and β2-microglobulin (used to visualize cell morphology) immunostaining. **C** Immunocytochemistry for extraembryonic cell markers (HLA-G, hCGA, GABRP, and ERVW-1) in the 4 types of h-Muse cells at 3 weeks after induction. **D** Western blot of hCGB, a functional marker of human trophoblasts, in the naïve state and 3 weeks after induction in h-UC-Muse cells. JEG3 was set as a positive control (P.C.). Bars: 50 μm. *p < 0.05, **p < 0.01, ***p < 0.001. *ND* not detected

In qPCR, ID2 (early trophoblast marker) [73] was expressed in all 4 types of naïve Muse cells, and TP63 (undifferentiated trophoblast cell marker) [74] was expressed in naïve-h-AT- and -DT- but not in naive-h-UC- and -h-BM-Muse cells. On the other hand, CDX2 (trophectoderm-specific transcription factor involved in trophectoderm formation) [75], PLGF (angiogenic factor involved in placenta formation) [76], ERVFRD-1 (also known as syncytin 2; involved in the fusion of cytotrophoblasts) [77], and GCM1 (placenta-specific transcription factor involved in the placenta development) [78, 79] were under the detection limit in the 4 types of naïve Muse cells (Fig. 4A).

As the induction progressed, all of these markers increased over time in h-UC-Muse cells, whereas CDX2, PLGF, ERVFRD-1, and GCM1 were consistently under the detection limit in h-BM-, h-AT-, and h-DT-Muse cells until 3 weeks (Fig. 4A). ID2 and TP63 increased over time in the 4 Muse cell types, reaching the maximum level at 2 weeks in h-UC-Muse cells and at 3 weeks in h-AT-Muse cells (Fig. 4A). In h-BM-Muse cells, ID2 and TP63 levels peaked at day 5, and in h-DT-Muse cells ID2 peaked at 2 weeks and TP63 peaked at 3 weeks (Fig. 4A). In contrast to the other 3 Muse cell types, h-UC-Muse cells newly expressed CDX2 and PLGF at day 3, and ERVFRD-1 and GCM1 at 1 week, with CDX2 peaking at 1 week, and PLGF, ERVFRD-1, and GCM1 peaking at 3 weeks (Fig. 4A).

Syncytiotrophoblast-like multinucleated cells were observed in h-UC-Muse cells, but not in h-BM-, h-AT-, or h-DT-Muse cells, at 3 weeks (Fig. 4B). In immunocytochemistry, cells positive for trophoblast markers such as HLA-G (mononucleated extravillous trophoblast marker) [80–82], human chorionic gonadotropin alpha (hCGA) [83], gamma-aminobutyric acid receptor subunit pi (GABRP) [84], and endogenous retrovirus group W envelope member 1 (ERVW-1, also known as syncytin 1 involved in trophoblast fusion) [85], were detected in h-UC-Muse cells but not in h-BM-, h-AT-, or h-DT-Muse cells (Fig. 4C, positive and negative controls for immunostaining are shown in Fig. S8A). In the Western blot, hCG beta (hCGB; functional marker synthesized by placental trophoblasts) [86] was under the detection limit in naïve-h-UC-Muse cells (Fig. 4D). This marker was newly expressed in h-UC-Muse cells at 3 weeks after induction (Fig. 4D).

### Germline-lineage marker expression by in vitro induction

Unlike primed PSCs, naïve PSCs can differentiate into germline lineages [63]. The 4 Muse cell types were treated with cytokines and reagents reported to induce iPS cells to differentiate into germline lineages with minor modifications [53].

**Fig. 5:**
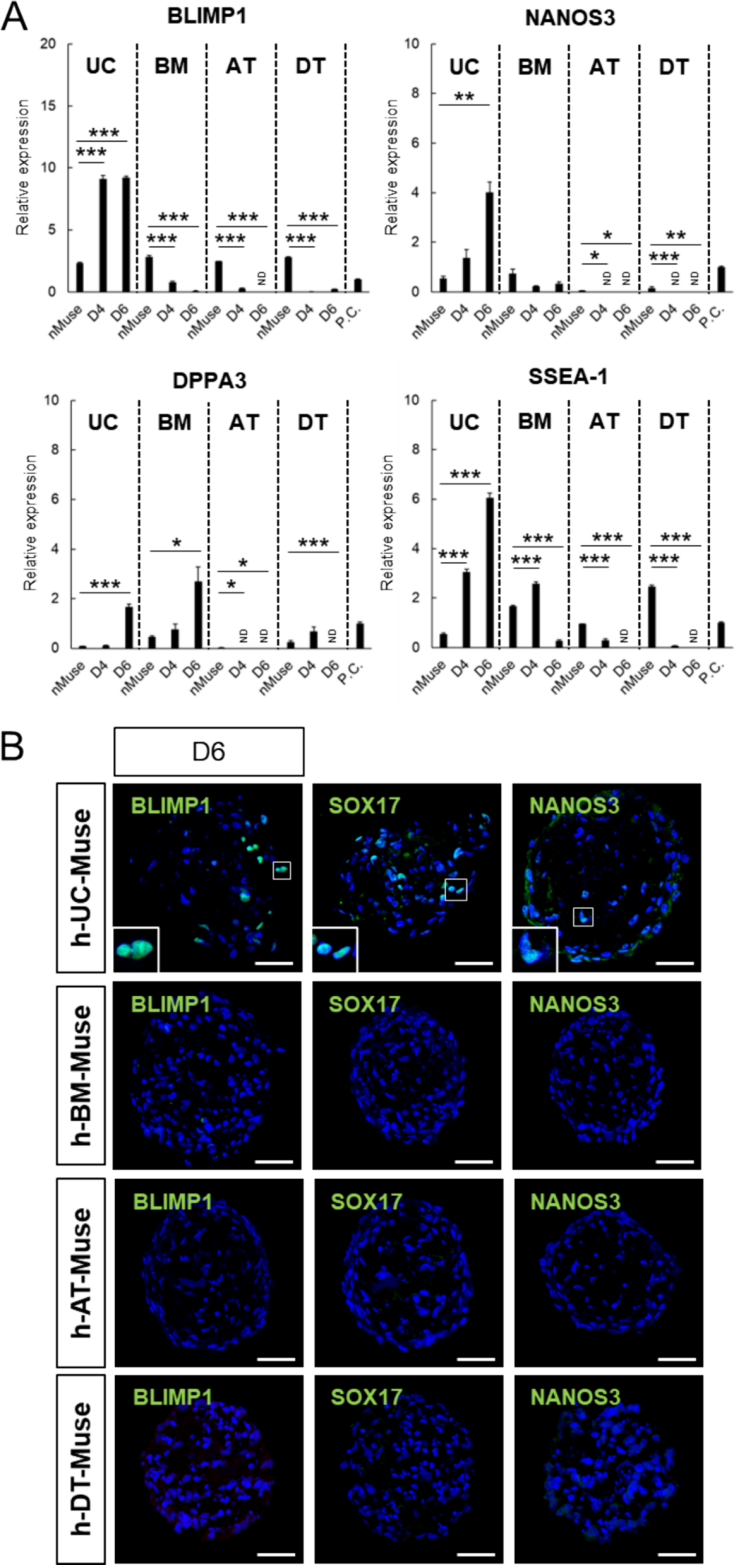
In vitro differentiation of h-UC-Muse cells into germline-lineage marker(+) cells. **A** Expression of genes related to germline lineage in the 4 types of naïve Muse cells, as well as in those from day 4 to day 6 after induction (normalized by ACTB). PGCLC was set as a positive control (P.C.). Values of PGCLC were set as 1. **B** Immunocytochemistry for germline markers (BLIMP1, SOX17, and NANOS3) in the 4 types of h-Muse cells at 3 weeks after induction. Bars: 50 μm. *p < 0.05, **p < 0.01, ***p < 0.001. *ND* not detected

In qPCR, transcription factors essential for primordial germ cell (PGC) formation, such as BLIMP1 [87] and NANOS3 [88], as well as transcription factors expressed in PGCs, such as DPPA3 [89, 90], and SSEA-1 (cell surface marker of PGCs) [91], were all expressed in the 4 types of naïve Muse cells (Fig. 5A).

As the induction progressed, all of these markers were increased in h-UC-Muse cells, while the other 3 types of Muse cells responded only partially; in h-BM-, h-AT-, and h-DT-Muse cells, the expression level of BLIMP1, NANOS3, and SSEA-1 at day 6 was lower than that in the naïve state and the expression level of DPPA3 became under the detection limit at day 6 except in h-BM-Muse cells (Fig. 5A).

Immunohistochemistry on day 6 showed the presence of cells positive for BLIMP1, SOX17, and NANOS3 in h-UC-Muse cells, while those markers were undetectable in the other 3 Muse cell types (Figs. 5B, S8B).

### Hematopoietic-lineage marker expression by in vitro induction

It is considered difficult to induce HSCs from naïve and primed PSCs [54, 92–97]. The induction requires multiple steps: PSCs should first be induced into the early mesoderm lineage, followed by differentiation into hemangioblasts (common precursor for HSCs and endothelial cells) and hemogenic endothelium, and finally into HSCs [54]. Referring to a previous report, the 4 Muse cell types were differentiated into HSCs with minor modifications [54].

In qPCR of the naïve state, the hemogenic endothelium marker TIE2 [98] was expressed in h-UC- and h-BM-Muse cells, but not in h-AT- and h-DT-Muse cells. In contrast, the hemangioblast/hemogenic endothelium marker FLK-1 (also known as vascular endothelial growth factor receptor 2) [99], was expressed in all types of naïve Muse cells except h-BM-Muse cells. The hemogenic endothelium marker CD31 [100, 101] was expressed in the 4 types of naïve Muse cells, and the hemogenic endothelium/HSC marker c-KIT [102] was expressed only in naïve h-UC-Muse cells. The hematopoietic stem/progenitor, cell marker CD34 [103] was detected in naïve h-UC- and h-BM-Muse cells. In contrast, hematopoietic lineage marker PTPRC (also known as CD45) [104] was under the detection level in all types of naïve Muse cells.

**Fig. 6:**
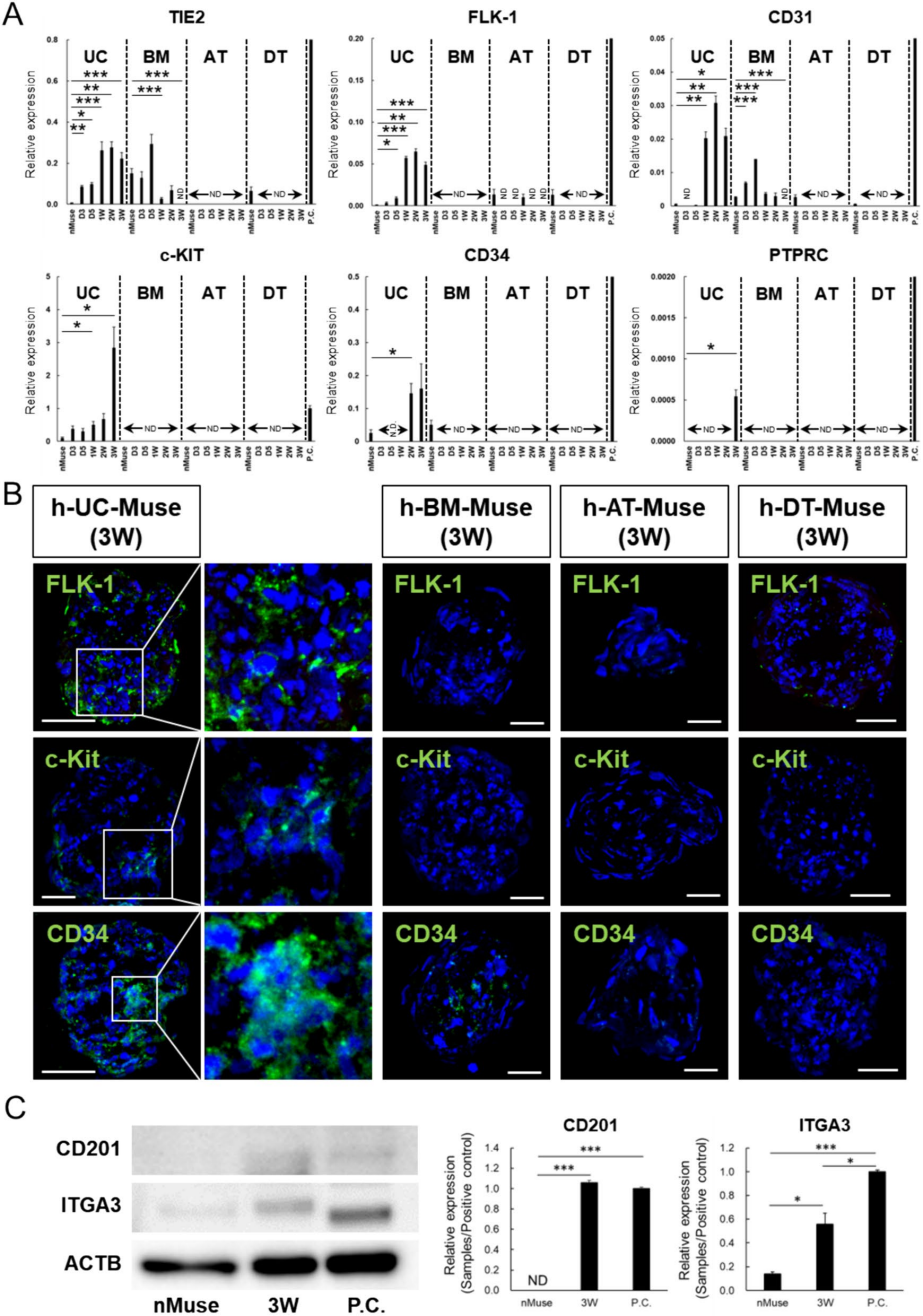
In vitro differentiation of h-UC-Muse cells into hematopoietic-lineage marker(+) cells. **A** Expression of genes related to the hematopoietic lineage in the 4 types of naïve Muse cells, as well as those from day 3 to 3 weeks after induction (normalized by ACTB). For the positive control, human fetus whole total RNA (TIE2, FLK-1, CD31, and PTPRC) and human blood total RNA (c-KIT and CD34) were used (P.C.). Values of PGCLC were set as 1. **B** Immunocytochemistry for FLK-1, c-Kit, and CD34 in the 4 types of h-Muse cells at 3 weeks after induction. **C** Western blot of CD201 and ITGA3 in the naïve state and 3 weeks after induction in h-UC-Muse cells. HeLa cells were set as a positive control (P.C.), according to previous reports [116, 117]. Bars: 50 μm. *p < 0.05, **p < 0.01, ***p < 0.001. *ND* not detected

As the induction progressed, all of the above markers were increased in h-UC-Muse cells with the maximum level TIE2, FLK-1, and CD31 at 2 weeks, and of c-KIT, CD34, and PTPRC at 3 weeks (Fig. 6A). In contrast, the other 3 Muse cell types responded partially; c-KIT, CD34, and PTPRC remained under the detection limit until 3 weeks; TIE, FLK-1, and CD31 became under the detection limit after day 3, and that was maintained until 3 weeks in h-BM-, h-AT-, and h-DT-Muse cells, except for TIE2 and CD31 in h-BM-Muse cells, which peaked at day 5 but declined after that; and sporadic expression of FLK-1 was observed in h-AT-Muse cells at 1 week (Fig. 6A).

In immunohistochemistry, cells positive for FLK-1, c-Kit, and CD34 were detected in h-UC-Muse cells but not in the other 3 Muse cell types at 3 weeks (Figs. 6B, S8C). In the Western blot, CD201 (functional marker involved in the maintenance of HSCs) [105] was under the detection limit in naïve-h-UC-Muse cells (Fig. 6C). In contrast, integrin subunit alpha 3 (ITGA3), another functional marker involved in the maintenance of HSCs) [106], was expressed in naïve-h-UC-Muse cells (Fig. 6C). In h-UC-Muse cells at 3 weeks after induction, CD201 was newly expressed, and ITGA3 was increased 4-fold compared with that in naïve-h-UC-Muse cells. (Fig. 6C).

## Discussion

### Similarities between h-UC-Muse cells and h-adult tissue-Muse cells

Human-UC-Muse cells, identified as SSEA-3(+) cells in both immunohistochemistry of the UC tissue and FACS analysis (Figs. 1A, S1A), expressed pluripotency markers such as Nanog, Oct3/4, Sox2, PAR4 and Tra-1-81 (Fig. 1B); differentiated into triploblastic-lineage cells at a single cell level (Fig. S3C); and were non-tumorigenic as supported by the low telomerase activity (Fig. 1C) and the absence of teratoma formation in vivo until 6 months (Figs. 1D, S3D) [1, 3, 6]. They also expressed many MSC markers and SSEA-4, a member of the globo series of glycosphingolipids. They were negative for hematopoietic markers CD34 and CD45, the neural crest stem cell marker CD271, and the vascular endothelial cell marker vWF (Fig. S2A). They also exhibited phagocytotic activity (Fig. S4A-C), selective homing to the damaged tissue by intravenous injection (Fig. 1F-G), and in vivo differentiation into tissue-specific cells in the homed tissue, as demonstrated in the CCl_4_-induced mouse acute liver injury model (Fig. S5) [60]. These characteristics are similar to those reported in h-adult tissue-Muse cells such as h-BM-, h-AT-, and h-DT-Muse cells [1–3, 9].

### Human-UC-Muse cells were more similar to h-post-implantation blastocysts than h-adult tissue-Muse cells

In h-UC-Muse cells, CD133 (a marker for ESCs, HSCs, NSCs, and cancer stem cells) and HLA-G (relevant to immunotolerance in the placenta) expression were as high as ~ 99%- and ~ 65%, respectively. At the same time, in h-adult tissue-Muse cells, CD133 and HLA-G were only expressed at several percent and 2 ~ 17%, respectively (Fig. 1A).

Among h-UC-Muse cells, ~ 20% of cells were XIST negative, a characteristic of naïve PSCs, while all of the analyzed h-BM-, h-AT- and h-DT-Muse cells were XIST positive (Fig. 1H).

In the scRNA-seq, h-UC-Muse cells showed a distinct gene expression pattern from that in h-adult tissue-Muse cells that was more similar to that of h-embryo cells (E3-7, E8-14, and E16). Consistently, h-UC-Muse cells expressed DSG2, DSC3, and PG, known to be expressed in early human embryos, at higher levels than expressed by h-adult tissue-Muse cells (Fig. 2H) [71, 72]. Human-UC-Muse cells and h-embryo cells had similar expression levels of genes related to ectodermal, mesodermal, endodermal, hematopoietic, germline, and extraembryonic lineages (Fig. 2G).

Human-UC-Muse cells were separated into 3 distinct clusters whose gene expression patterns resembled that of h-post-implantation blastocysts comprised of h-epiblast and h-trophectoderm cells. (Fig. 2C, D). HLA-G is expressed in extravillous trophoblast cells derived from the trophectoderm of the blastocyst [80–82]. HLA-G was expressed at a higher ratio in h-UC-Muse cells than in h-adult tissue-Muse cells (Fig. 1A).

The overall DNA methylation level did not essentially differ among the 4 Muse cell types: h-UC-, h-BM-, h-AT, and h-DT-Muse cells. The DNA methylation level in the h-post-implantation blastocysts was slightly higher than that in the 4 Muse cell types, but the DNA methylation pattern in the h-post-implantation blastocysts was more similar to that in h-UC-Muse cells than to that in the other 3 Muse cell types (Fig. 3A, B and Fig. S7A-B). The repertoire of hypomethylated and hypermethylated genes, however, was interesting. In both h-UC-Muse cells and post-implantation blastocysts, genes related to differentiation, cell growth, and signaling pathways were more hypomethylated than those in h-adult tissue-Muse cells, and genes related to the immune response were more hypermethylated than those in h-adult tissue-Muse cells (Fig. 3C–E). Consistently, the heatmap showed a lower methylation level of genes related to ectodermal, mesodermal, endodermal, hematopoietic, germline, and extraembryonic lineages in h-UC-Muse cells than in h-adult tissue-Muse cells (Fig. 3F).

The gene expression pattern related to extraembryonic, germline, and hematopoietic lineages in h-UC-Muse cells was similar to that of h-post-implantation blastocysts, as suggested by scRNA-seq and DNA methylation status. In vitro differentiation induction showed the clear difference between h-UC-Muse cells and h-adult tissue-Muse cells; in qPCR, h-UC-Muse cells newly expressed markers specific to extraembryonic, germline, and hematopoietic lineages after induction, and markers were both already expressed at the naïve state and newly expressed after induction were elevated according to the time course. Human-adult tissue-Muse cells, however, responded partially or sporadically to the induction. Immunohistochemical analysis supported these results.

### Unique characteristics of h-UC-Muse cells

Human-UC-Muse cells are a heterogeneous population, as suggested by several results. In the surface marker expression, ~ 65% of the cells were positive for HLA-G, and ~ 62% for CD29 (Figs. 1A, S2A), suggesting the presence of cells negative for those markers. The presence of ~ 20% of XIST-negative cells means that ~ 80% of h-UC-Muse cells are XIST-positive. Notably, the UMAP analysis in scRNA-seq revealed that h-UC-Muse cells are primarily divided into 3 clusters: clusters 1, 3, 7 that were similar to h-post-implantation blastocysts, more precisely, epiblast and trophectoderm cells.

PSCs are generally classified into 2 phases: naive and primed [63]. Naive PSCs resemble the pluripotency of the inner cell mass before implantation and can differentiate into triploblastic cells and extraembryonic cells, germline, and hematopoietic lineages. Their DNA methylation levels are low, and both X chromosomes are transcriptionally active. On the other hand, primed PSCs resemble the pluripotency of post-implantation epiblasts, which can differentiate not only into triploblastic cells but also into extraembryonic, germline, and hematopoietic lineages. Their DNA methylation levels and histone modifications related to transcription repression are high, and either 1 of the 2 X chromosomes is inactivated via XIST. Therefore, h-UC-Muse cells are suggested to comprise the XIST-negative-subpopulation, more like naïve PSCs, and the XIST-positive major population, more like h-post-implantation epiblasts, namely primed PSCs.

In humans, 1 of the 2 X chromosomes is inactivated in the epiblast of the blastocyst after implantation, and this inactivation is maintained throughout development [107–111]. Consistent with developmental stages, naïve PSCs exhibit the characteristic of pre-X chromosome inactivation, while primed PSCs show post-X chromosome inactivation [111]. XIST expression was observed in all the observed h-BM-, h-AT-, and h-DT-Muse cells, indicating inactivation of 1 X chromosome (Fig. 1H). Because the h-UC-Muse cells used in this study were collected from h-UC-MSCs at passages 7 – 10, the characteristics of original h-UC-Muse cells are unknown. During passages, the original characteristics of Muse cells, such as XIST expression, gene expression profile, and DNA methylation state, will change.

The findings of the present study suggested that h-UC-Muse cells differentiated not only into triploblastic cells, but also into extraembryonic, germline, and hematopoietic lineages, thus exhibiting a broader differentiation potential than h-adult tissue-Muse cells (Figs. 4, 5 and 6). Human-adult tissue-Muse cells did not display such differentiation changes, and some extraembryonic, germline, and hematopoietic markers were under the detection limit after induction. Regarding extraembryonic induction, the factors involved in the fusion of cytotrophoblasts, such as ERVFRD-1 and ERVW-1, were expressed only in h-UC-Muse cells, suggesting the presence of syncytiotrophoblast-like multinucleated cells producing hCG. As for hematopoietic induction, the expression of CD201 and ITGA3 correlates with HSC activity. The presence of functional markers for CD201 and ITGA3 in h-UC-Muse cells at 3 weeks after induction suggests differentiation into more functional HSC-like cells. Interestingly, CD201 is highly expressed in fetal liver HSCs and cord blood HSCs while minimally expressed in BM-HSCs [112]. Although further research is necessary, cells differentiated from h-UC-Muse cells might partially resemble fetal liver HSCs and cord blood HSCs. Although the differentiation potential into extraembryonic, germline, and hematopoietic lineages was demonstrated, it is unclear which of the above 3 clusters (clusters 1, 3, 7) of h-UC-Muse cells played the central role.

Among various stem/progenitor cells in living bodies, those that exhibit properties similar to early human developmental stages, such as post-implantation blastocysts, have not yet been found. In this context, h-UC-Muse cells isolated as SSEA-3(+) from easily accessible h-UC without ethical issues are attractive cell sources as research tools.

### Potential of h-UC-Muse cells for clinical application

MSCs harvested from adult tissues, such as the BM, adipose tissue, and skin, have been used in various clinical trials but invasive procedures are required for the collection of MSCs. In contrast, fetal appendages like the UC can be collected noninvasively and are usually disposed of as medical waste. In this study, ~ 10 g UC tissue yielded ~ 1 × 10^7^ cells of h-UC-Muse cells as SSEA-3(+) cells. Based on calculations from this study, we estimate that ~ 5 × 10^7^ cells can be obtained from ~ 50 g UC tissue from a term pregnancy within 3 weeks. In comparison, ~ 20 mL of the BM would be required for collecting an equivalent number of SSEA-3(+) cells [1, 113], making the UC a realistic cell source for basic research and clinical application with few safety and ethical issues. Human-UC-Muse cells were superior to h-BM-Muse cells, the most generally used Muse cell type for preclinical and clinical research, in migrating to the injured site after intravenous injection, as demonstrated in the mouse acute liver damage model. This might be partly explained by the higher gene expression level of genes related to cell migration, such as BDKRB1, F3, CORO1A, and S1P receptor in h-UC-Muse cells than in h-BM-Muse cells.

HLA-G, which plays a vital role in immunosuppression in the placenta, was expressed in higher ratio in h-UC-Muse cells than in h-BM-Muse cells. This is expected to make UC-Muse cells beneficial for clinical treatment in terms of escaping immunologic rejection, which may lead to a higher engraftment ratio in damaged tissue.

## Limitations

This study has some limitations. Evaluation of the differentiation potential into extraembryonic, germline, and hematopoietic lineages was limited to assessing marker expression in vitro, representing only a partial and preliminary stage. Therefore, further research is necessary to determine whether h-UC-Muse cells have the ability to differentiate into those lineages in vivo.

Due to the difficulty of DNA methylation analysis in h-embryo cells, we used the h-embryo data (GSE49828) in this study. Because we compared data analyzed using different methodologies, there are limitations to the data comparison [114]. Specifically, while the C to T conversion rate was comparable between h-embryo cells and h-Muse cells, the mapping ratio was lower in h-embryo cells, and the mean coverage and total unique CpG sites were lower in h-Muse cells (Supplemental Table 4). The lower mapping rate in h-embryo cells is likely due to the analysis based on a significantly smaller number of cells (~ 200 cells) than Muse cells (1 × 105 cells). Regarding mean coverage and total unique CpG sites, Muse cells were analyzed using enzymatic methyl sequencing, mapping reads across the entire genome. In contrast, the h-embryo data were analyzed using RRBS, which concentrated reads in CpG-rich regions. Therefore, when calculating the average coverage and depth for regions where reads are mapped, RRBS showed higher values due to the focus on CpG-rich regions. The methylation correlation matrix was based on a relatively small fraction of the CpGs in the genome that were represented at > 10 × coverage. Further research is needed to comprehensively understand the DNA methylation status of h-embryo cells and h-Muse cells.

Placentas from term pregnancies exhibit widespread sex differences in hormone signaling, immune signaling, and metabolic functions [115]. The SSEA-3-positivity of h-UC-Muse cells from term pregnancies was similar between males and females (Supplemental Table 3). On the other hand, while the expression of pluripotency markers in h-UC-Muse cells varied between different lots, no clear sex differences were observed, likely due to the small number of samples analyzed (Fig. S3A). More detailed analysis is essential to fully understand potential sex differences in h-UC-Muse cells.

### Supplementary Information

Below is the link to the electronic supplementary material.

Supplementary file1 (DOCX 5606 KB)

Supplementary file2 (DOCX 17 KB)

Supplementary file3 (DOCX 16 KB)

Supplementary file4 (DOCX 16 KB)

Supplementary file5 (DOCX 18 KB)

Supplementary file6 (MP4 23421 KB)

## Data Availability

All the data is contained within the article and supporting information and will be available upon request.
