# Supplementary material for: Human post-implantation blastocyst-like characteristics of Muse cells isolated from human umbilical cord
Source: Cell Mol Life Sci. 2024 Jul 11;81(1):297. doi: 10.1007/s00018-024-05339-4 (PMC11335221; doi:10.1007/s00018-024-05339-4)
Supplement: Supplementary file 1 — Supplementary file1 (DOCX 5606 KB) [file 18_2024_5339_MOESM1_ESM.docx]

**
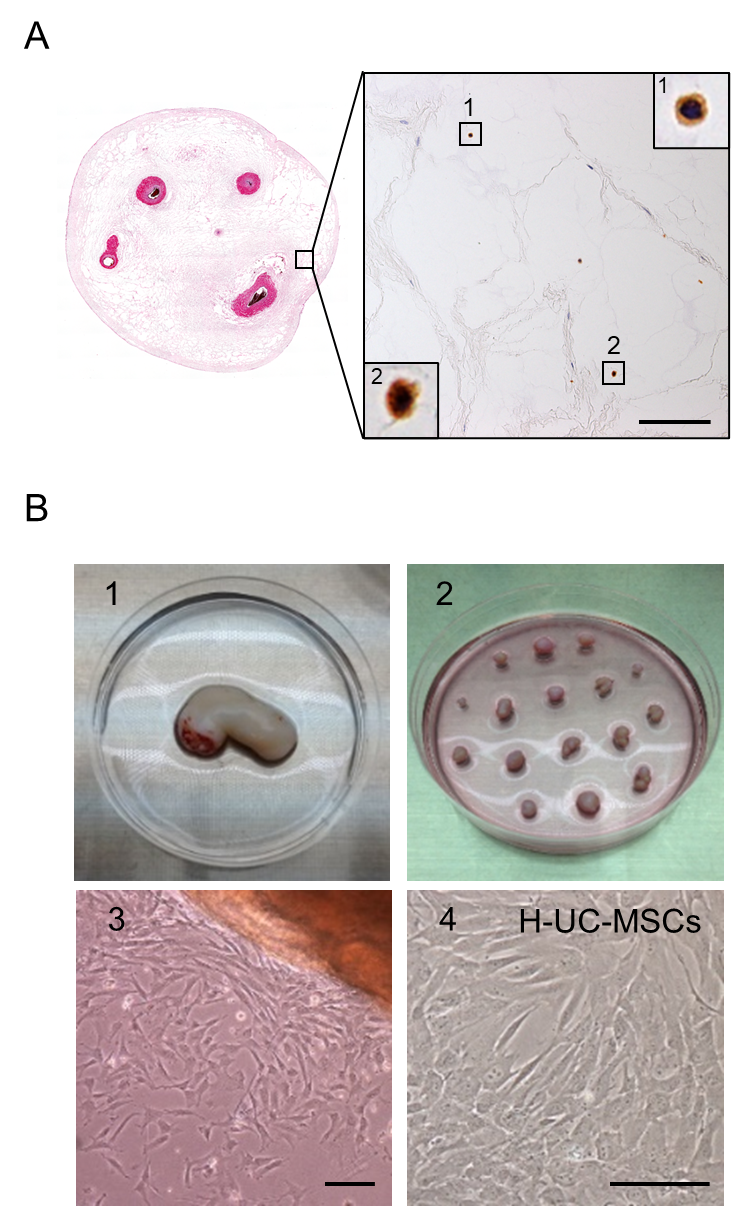
**

**Supplementary Figure 1.**

(A) (Left) H&E staining of h-UC and (Right) immunohistochemistry of SSEA-3(+) cells in the boxed area (Bar: 100 µm).

(B) The h-UC-MSC isolation procedure. The UC was minced into ~5 mm^3^ pieces, subsequently plated on dishes, and cultured for approximately 2 weeks to isolate adherent cells (Bars: 50 µm).


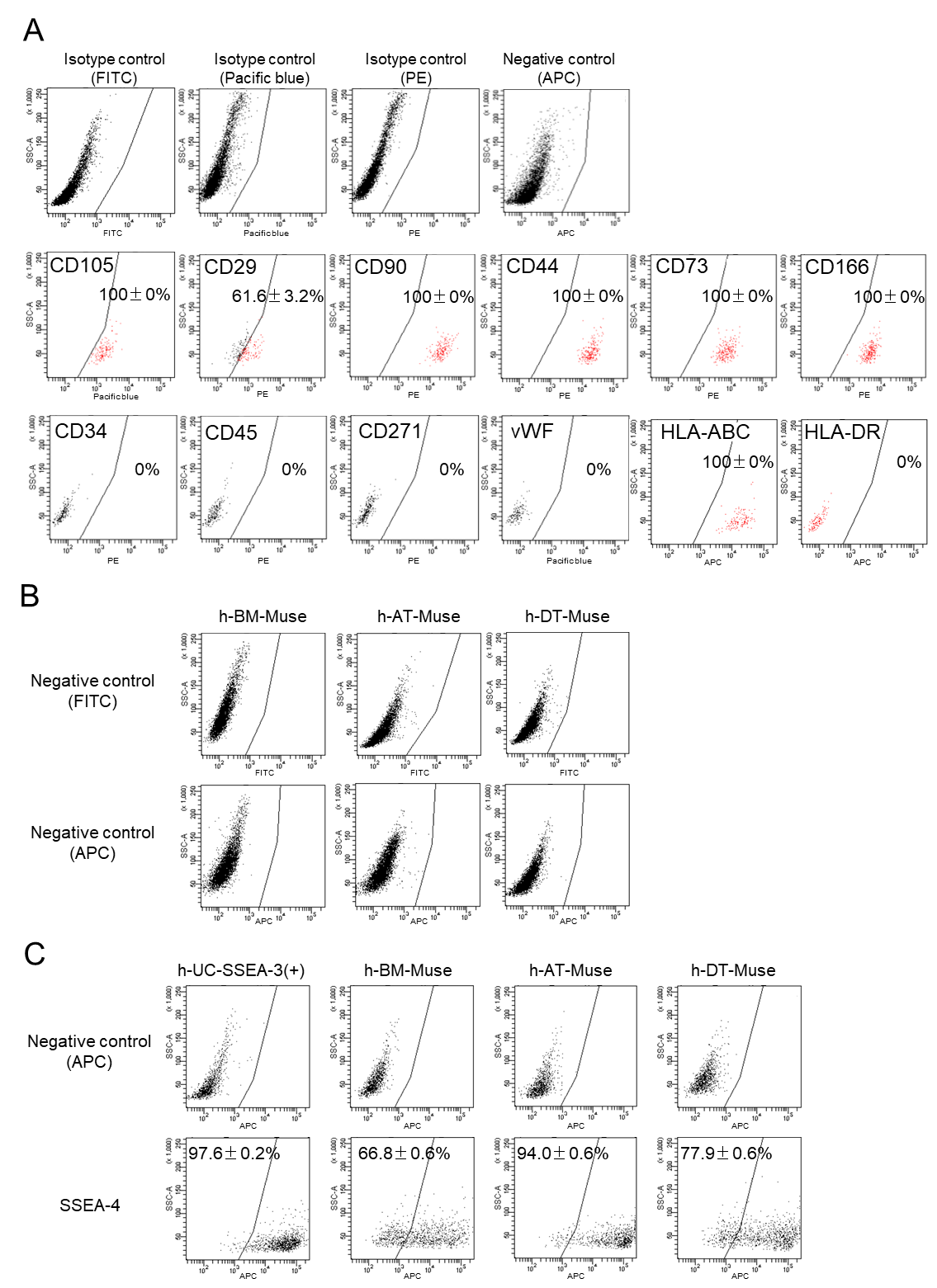


**Supplementary Figure 2.**

(A) Expression of mesenchymal markers (CD105, CD29, CD90, CD44, CD73, and CD166), hematopoietic markers (CD34 and CD45), neural crest stem cell marker (CD271), vascular endothelial cell marker (von Willebrand factor [vWF]), HLA-ABC, and HLA-DR in h-UC-SSEA-3(+) cells (mean ± SEM).

(B) Negative control using FITC- or APC-conjugated secondary antibody in h-BM-, h-AT-, and h-DT-Muse cells.

(C) Expression of SSEA-4 in h-UC-, h-BM-, h-AT-, and h-DT-Muse cells (mean ± SEM).

**
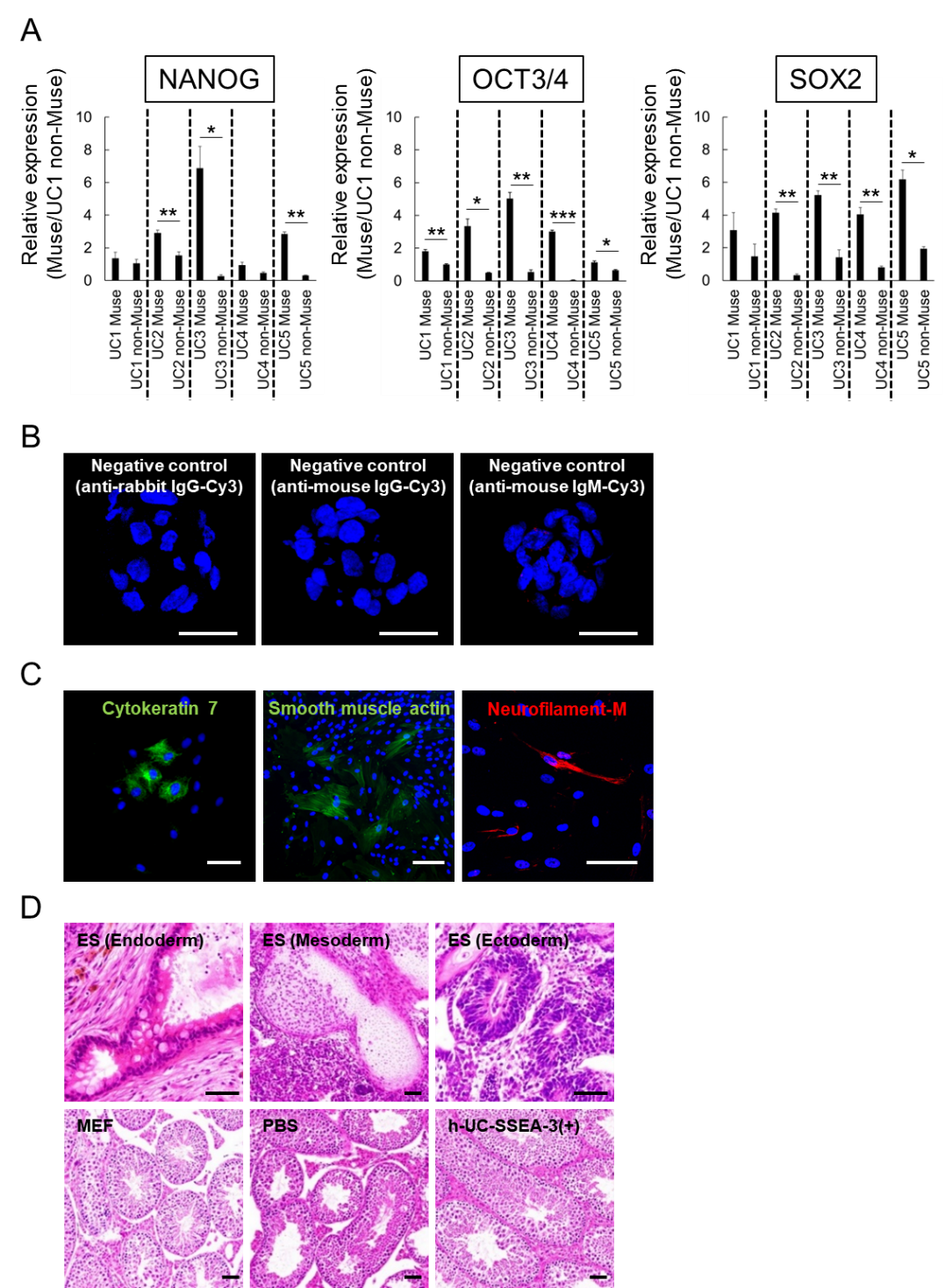
**

**Supplementary Figure 3.**

(A) Expression of pluripotency genes in h-UC-SSEA-3 (+) and SSEA-3(-) cells isolated from h-UC1-5 (mean ± SEM). *p < 0.05, **p < 0.01, ***p < 0.001.

(B) Negative control for each pluripotent marker (Bars: 50 µm).

(C) Immunocytochemistry for cytokeratin 7, smooth muscle actin, and neurofilament-M in cells derived from a single SSEA-3(+)-derived cluster. The cluster was transferred to a gelatin-coated adherent culture dish to allow the cells to expand from the cluster (Bars: 50 µm).

(D) H&E staining. Formation of teratomas in the SCID mouse testis that received mouse ES cell injection at 3 months. The teratoma contained endodermal, mesodermal, and ectodermal tissues. Injection of MEF, PBS, and h-UC-SSEA-3(+) cells that did not form teratomas after 6 months maintained normal testicular structure (Bars: 50 µm).

**
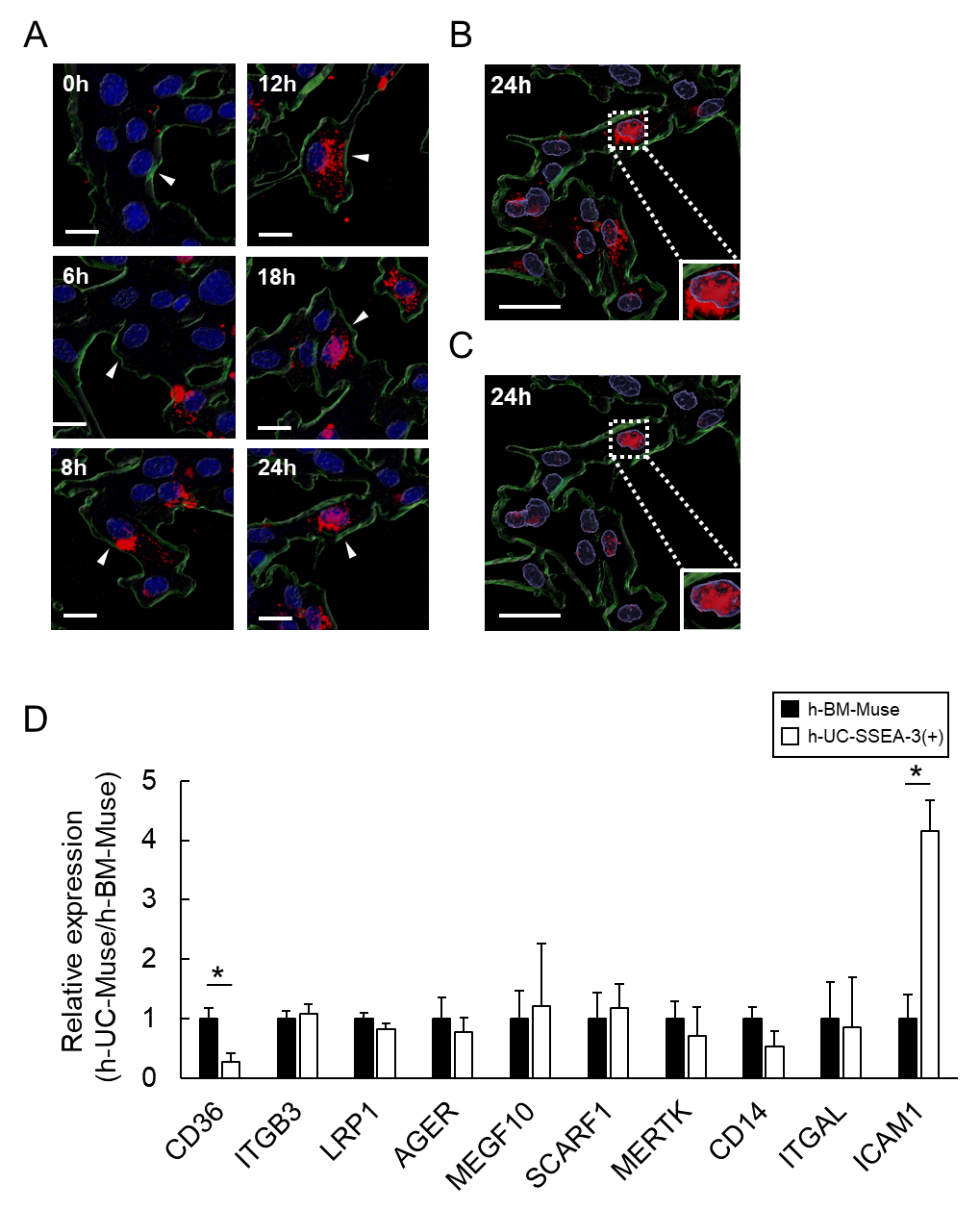
**

**Supplementary Figure 4.**

(A) Laser confocal microscopy of GFP-h-UC-Muse cells incubated with dead cell fragments collected from rotenone- and antimycin A-treated mCherry-labeled Hepa1-6 for 24 h (see Movie 1).

(B) Live imaging at 24 h with selective extraction of intracellular mCherry signal, as visualized by Imaris software.

(C) Analysis of (B) using Imaris software selectively extracted the mCherry signal within the nucleus.

(D) Gene expression of each phagocytosis receptor in h-BM-Muse cells and h-UC-SSEA-3(+) cells based on next generation sequencing data. The relative expression level of phagocytosis receptors in h-UC-SSEA-3(+) cells and h-BM-Muse cells was calculated with the average h-BM-Muse cells as the reference (mean ± SEM) value. *p < 0.05.

**
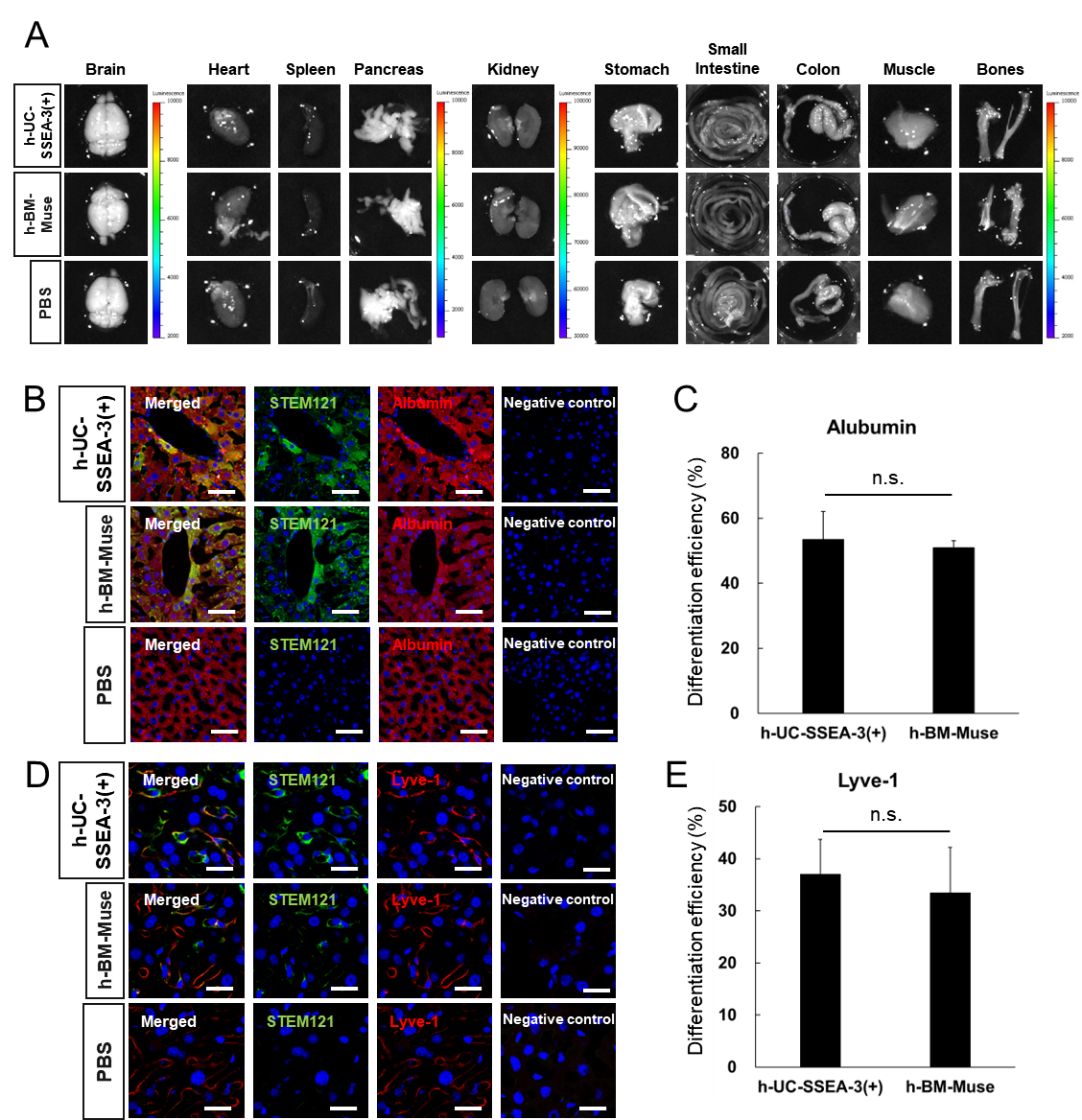
**

**Supplementary Figure 5.**

(A) In vivo dynamics of intravenously injected h-UC-SSEA-3(+) cells, h-BM-Muse cells and PBS in acute liver injury model. The Akaluc signal in the organs displayed in this figure was under the detection limit.

(B) Immunofluorescence for STEM121 (green), albumin (red), and DAPI (blue) in the h-UC-SSEA-3(+)-, h-BM-Muse- and PBS-groups (Bars: 50 µm).

(C) The percent of albumin(+) cells for all the STEM121(+) cells.

(D) Immunofluorescence for STEM121 (green), lyve-1 (red) and DAPI (blue) in the h-UC-SSEA-3(+)-, h-BM-Muse- and PBS-groups (Bars: 25 µm).

(E) The percent of lyve-1 (+) cells to the total STEM121(+) cells.


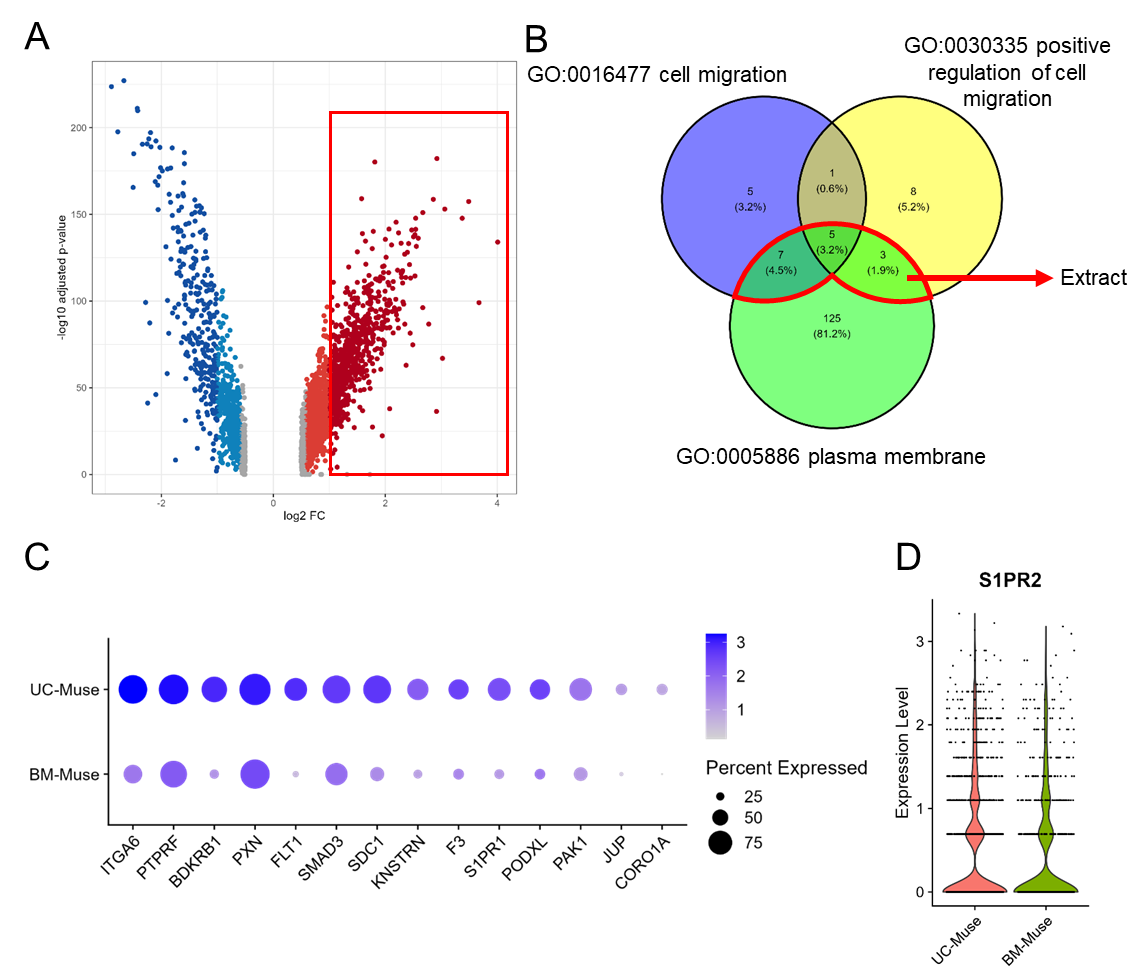


**Supplementary Figure 6.**

(A) Volcano plot of scRNA-seq data displaying the difference in the gene expression pattern between h-UC-Muse cells and h-BM-Muse cells. Genes with a fold change greater than 1.5 and adjusted p value less than 0.05 in h-UC-Muse cells compared to h-BM-Muse cells were extracted.

(B) The identified genes were common to any of 3 GO terms related to cell migration (cell migration, positive regulation of cell migration, and positive regulation of cell migration).

(C) Dot plots of gene expression involved in cell migration. Color gradient of dots represents the expression level, while the dot size indicates the percent of cells in each group.

(D) Violin plot showing the expression levels of S1PR2 in h-UC-, h-BM-Muse cells.


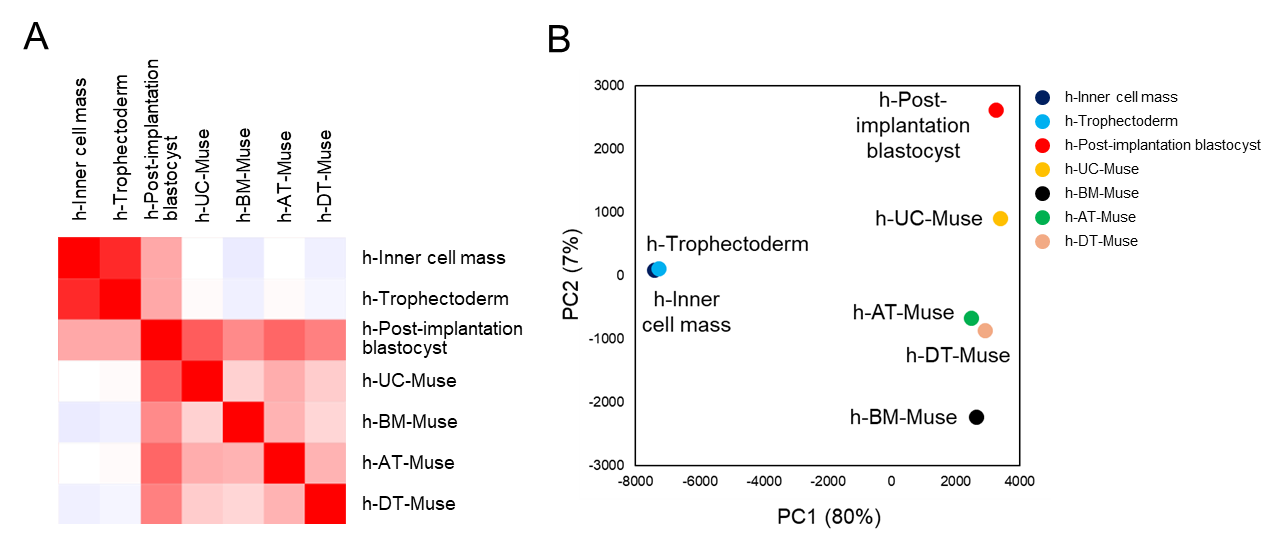


**Supplementary Figure 7.**

(A) Correlation matrix showing Pearson’s correlation coefficients between h-Muse cells and h-embryo cells.

(B) Principal component analysis (PCA) plot of h-Muse cells and h-embryo cells.


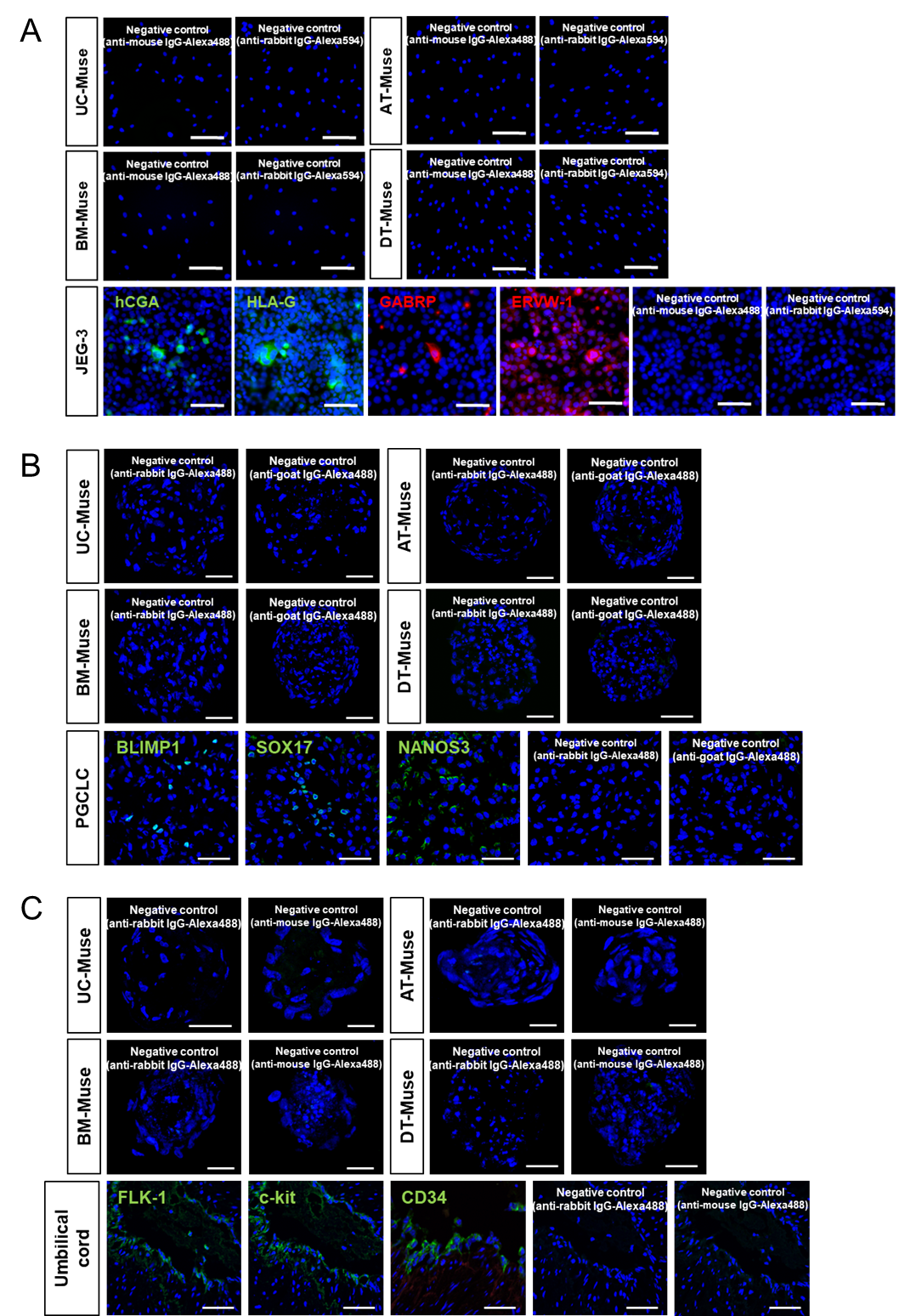


**Supplementary Figure 8.**

(A) Positive and negative controls for extraembryonic-lineage markers (Bars: 50 μm).

(B) Positive and negative controls for germline-lineage markers (Bars: 50 μm).

(C) Positive and negative controls for hematopoietic-lineage markers (Bars: 50 μm).
