# Supplementary material for: Human post-implantation blastocyst-like characteristics of Muse cells isolated from human umbilical cord
Source: Cell Mol Life Sci. 2024 Jul 11;81(1):297. doi: 10.1007/s00018-024-05339-4 (PMC11335221; doi:10.1007/s00018-024-05339-4)
Supplement: Supplementary file 2 — Supplementary file2 (DOCX 17 KB) [file 18_2024_5339_MOESM2_ESM.docx]

Supplementary Table 1. List of primary antibodies

| **Antibodies** | **Supplier** | **Catalog number** | **Concentration** |
| --- | --- | --- | --- |
| Purified rat anti-human/mouse SSEA-3 | Thermo Fisher Scientific | MA1-020 | 1:200 |
| Purified rat anti-human/mouse SSEA-3 | BioLegend | 330302 | 1:1000 |
| Purified rat IgM Isotype Ctrl | BioLegend | 400801 | 1:1000 |
| Allophycocyanin (APC)-labeled mouse anti-human HLA-ABC | BioLegend | 311409 | 1:100 |
| APC-mouse anti-human HLA-DR | BioLegend | 307609 | 1:100 |
| Phycoerythrin (PE)-labeled mouse anti-human CD29 | BD | 556049 | 1:100 |
| PE-labeled mouse anti-human CD34 | BD | 555822 | 1:100 |
| PE-labeled mouse anti-human CD44 | BD | 550989 | 1:100 |
| PE-labeled mouse anti-human CD45 | BD | 555483 | 1:100 |
| PE-labeled mouse anti-human CD73 | BD | 550257 | 1:100 |
| PE-labeled mouse anti-human CD90 | BD | 555596 | 1:100 |
| PE-labeled mouse anti-human CD166 | BD | 559263 | 1:100 |
| PE-labeled mouse anti-human CD271 | BD | 557196 | 1:100 |
| Purified mouse anti-human CD105 | BD | 555690 | 1:100 |
| Purified mouse anti-human von Willebrand factor (vWF) | BD | 555849 | 1:100 |
| Purified mouse anti-human CD133 | Miltenyi Biotec | 130-090-422 | 1:100 |
| Purified mouse anti-human SSEA-4 | BioLegend | 330401 | 1:100 |
| Purified mouse anti-HLA-G | BD | 557577 | 1:50 |
| Mouse monoclonal antibody specific for human cytoplasmic marker (STEM121) | TaKaRa Bio Inc | Y40410 | 1:100 |
| Goat anti-albumin | Bethyl Laboratories | A80-229A | 1:100 |
| Rabbit anti-Lyve-1 | Novus Biologicals | NB600-1008 | 1:100 |
| Rabbit anti-Nanog | Millipore | AB5731 | 1:100 |
| Rabbit anti-Oct3/4 | Santa Cruz Biotechnology | sc-9081 | 1:100 |
| Rabbit anti-Sox2 | Millipore | AB5603 | 1:1000 |
| Mouse anti-PAR4 | Santa Cruz Biotechnology | sc-1666 | 1:100 |
| **Antibodies** | **Supplier** | **Catalog number** | **Concentration** |
| Mouse anti-TRA-1-81 | Santa Cruz Biotechnology | sc-21706 | 1:100 |
| Mouse anti-human chorionic gonadotropin alpha (hCGA) | R&D Systems | MAB4169 | 1:100 |
| Rabbit anti-gamma-aminobutyric acid A receptor, pi (GABRP) | aviva systems biology | AVARP13034_P050 | 1:100 |
| Rabbit anti-endogenous retrovirus group W member 1, envelope (ERVW-1) | Abcam | ab71115 | 1:100 |
| Rabbit anti-B lymphocyte-induced maturation protein 1 (BLIMP1) | Abcam | ab198287 | 1:100 |
| Goat anti-SOX17 | R&D Systems | AF1924 | 1:50 |
| Rabbit anti-Nanos C2HC-type zinc finger 3 (NANOS3) | Abcam | ab70001 | 1:100 |
| Mouse anti-SSEA-1 | Thermo Fisher Scientific | 41-1200 | 1:100 |
| Mouse anti-fetal liver kinase 1 (FLK-1) | Abcam | ab9530 | 1:100 |
| Rabbit anti-c-Kit | Abcam | Ab5506 | 1:50 |
| Mouse anti-CD34 | Santa Cruz Biotechnology | sc-7324 | 1:50 |
