# Supplementary material for: Human post-implantation blastocyst-like characteristics of Muse cells isolated from human umbilical cord
Source: Cell Mol Life Sci. 2024 Jul 11;81(1):297. doi: 10.1007/s00018-024-05339-4 (PMC11335221; doi:10.1007/s00018-024-05339-4)
Supplement: Supplementary file 3 — Supplementary file3 (DOCX 16 KB) [file 18_2024_5339_MOESM3_ESM.docx]

Supplementary Table 2. List of secondary antibodies

| **Antibodies** | **Supplier** | **Catalog number** | **Concentration** |
| --- | --- | --- | --- |
| Fluorescein isothiocyanate (FITC)-conjugated anti-rat IgM | Jackson ImmunoResearch Laboratories | 112-095-075 | 1:100 |
| Pacific Blue-labeled goat anti-mouse IgG | Thermo Fisher Scientific | P31582 | 1:100 |
| APC-labeled goat anti-mouse IgG | Jackson ImmunoResearch Laboratories | 115-136-146 | 1:100 |
| APC-conjugated goat anti-rat IgM | Jackson ImmunoResearch Laboratories | 112-136-075 | 1:100 |
| horse-radish peroxidase (HRP)-conjugated goat anti-rat IgM | Jackson ImmunoResearch Laboratories | 112-035-075 | 1:200 |
| Alexa Fluor 488-conjugated donkey anti-mouse IgG | Jackson ImmunoResearch Laboratories | 715-546-150 | 1:200 |
| Alexa Fluor 488-conjugated donkey anti-rabbit IgG | Jackson ImmunoResearch Laboratories | 711-546-152 | 1:200 |
| Alexa Fluor 488-conjugated donkey anti-goat IgG | Jackson ImmunoResearch Laboratories | 705-545-003 | 1:200 |
| Alexa Fluor 594-conjugated donkey anti-rabbit IgG | Jackson ImmunoResearch Laboratories | 711-586-152 | 1:200 |
| Cyanine-3 (Cy3)-conjugated donkey anti-mouse IgG | Jackson ImmunoResearch Laboratories | 715-167-003 | 1:200 |
| Cy3-conjugated goat anti-mouse IgM | Jackson ImmunoResearch Laboratories | 115-116-075 | 1:200 |
| Cy3-conjugated donkey anti-rabbit IgG | Jackson ImmunoResearch Laboratories | 711-167-003 | 1:200 |
