# Supplementary material for: Human post-implantation blastocyst-like characteristics of Muse cells isolated from human umbilical cord
Source: Cell Mol Life Sci. 2024 Jul 11;81(1):297. doi: 10.1007/s00018-024-05339-4 (PMC11335221; doi:10.1007/s00018-024-05339-4)
Supplement: Supplementary file 4 — Supplementary file4 (DOCX 16 KB) [file 18_2024_5339_MOESM4_ESM.docx]

Supplementary Table 3. Summary of specimens, obtained h-UC-MSCs and Muse cells

|  | Sex | Gestational age (wk) | SSEA-3 (+) cells in UC-MSCs (%) |
| --- | --- | --- | --- |
| h-UC1 | Male | 36 | 5.5 |
| h-UC2 | Female | 36 | 5.3 |
| h-UC3 | Male | 35 | 4.5 |
| h-UC4 | Female | 36 | 4.5 |
| h-UC5 | Female | 38 | 6.0 |
|  |  |  |  |
| Mean (SE) |  | 36.2 (0.5) | 5.2 (0.3) |
