# Supplementary material for: Human post-implantation blastocyst-like characteristics of Muse cells isolated from human umbilical cord
Source: Cell Mol Life Sci. 2024 Jul 11;81(1):297. doi: 10.1007/s00018-024-05339-4 (PMC11335221; doi:10.1007/s00018-024-05339-4)
Supplement: Supplementary file 5 — Supplementary file5 (DOCX 18 KB) [file 18_2024_5339_MOESM5_ESM.docx]

| Supplementary Table 4. Summary of the sequencing qualities, read mapping, and the covered CpG sites with their mean coverage depths at 1×, 5×, and 10×, as well as the C to T conversion rate in each sample. | | | | | | | | | | | |
| --- | --- | --- | --- | --- | --- | --- | --- | --- | --- | --- | --- |
| Sample | No. of Total Sequencing Reads | No. of Clean Reads After Trimming | No. of Mappable Reads | Mapping Ratio (%) | Total Unique CpG Sites(1×) | Mean Coverage(1×) | Total Unique CpG Sites(5×) | Mean Coverage(5×) | Total Unique CpG Sites(10×) | Mean Coverage(10×) | C to T Conversion Rate (%) |
| h-Inner cell mass | 187549610 | 168157254 | 76959784 | 45.8 | 5909713 | 48.0 | 3794533 | 72.2 | 2923665 | 89.4 | 98.9 |
| h-Trophectoderm | 130544004 | 120702996 | 50788460 | 42.1 | 5836671 | 32.3 | 3239166 | 54.5 | 2352024 | 68.9 | 99.0 |
| h-Post-implantation blastocyst | 182693174 | 164863298 | 89730754 | 54.4 | 8744266 | 39.3 | 6217024 | 53.1 | 5087412 | 59.9 | 99.4 |
| h-UC-Muse | 229372397 | 138491060 | 117424274 | 84.8 | 23225320 | 4.2 | 9375181 | 7.1 | 1071869 | 13.8 | 99.5 |
| h-BM-Muse | 249333569 | 145956381 | 116152176 | 79.6 | 39980087 | 4.0 | 15537084 | 6.9 | 1366700 | 14.1 | 96.2 |
| h-AT-Muse | 310158092 | 190507930 | 160648124 | 84.3 | 44067566 | 5.5 | 25925913 | 7.9 | 5537086 | 12.8 | 98.5 |
| h-DT-Muse | 202469115 | 115551960 | 88672463 | 76.7 | 37245562 | 3.2 | 9486603 | 6.7 | 545238 | 16.9 | 96.6 |
